# S100A9 Integrates Autophagic Deficiency With Immunopathology and Latanoprost Responsiveness in Primary Open‐Angle Glaucoma

**DOI:** 10.1155/ijog/3168725

**Published:** 2026-03-11

**Authors:** Lili Hu, Shaoxin Pan, Gong Chen, Min Lei, Ming Ai, Xiangyun Lv, Haoning Pan, Peng Wang, Rui Chang

**Affiliations:** ^1^ Ophthalmology Medical Center, The First Affiliated Hospital of Chongqing Medical University, Chongqing Key Laboratory for the Prevention and Treatment of Major Blinding Eye Diseases, Chongqing Branch (Municipality Division) of National Clinical Research Centre for Ocular Diseases, Chongqing, 400016, China, cqmu.edu.cn; ^2^ Ophthalmology Medical Center, The Second Affiliated Hospital of Wannan Medical College, Wuhu, Anhui, 241000, China, wnmc.edu.cn; ^3^ Cancer Medical Center, Renmin Hospital of Wuhan University, Wuhan, Hubei, 430060, China, rmhospital.com; ^4^ Ophthalmology Medical Center, Renmin Hospital of Wuhan University, Wuhan, Hubei, 430060, China, rmhospital.com; ^5^ Ophthalmology Medical Center, The Affiliated Tongren Hospital of Wuhan University, Wuhan, Hubei, 430060, China; ^6^ School of Clinical Medicine, Wannan Medical College, Wuhu, Anhui, 241002, China, wnmc.edu.cn

**Keywords:** autophagy, immune dysregulation, molecular docking, primary open-angle glaucoma, S100A9, trabecular meshwork

## Abstract

Primary open‐angle glaucoma (POAG) is a progressive optic neuropathy that leads to irreversible vision loss, primarily due to dysfunction of the trabecular meshwork (TM). Although impaired autophagy has been implicated in POAG pathogenesis, its molecular drivers remain poorly defined. This study systematically investigated autophagy‐related genes (ATGs) in TM tissue from POAG patients. Transcriptomic datasets (GSE4316 and GSE27276) were analyzed to identify differentially expressed genes (DEGs). A curated list of autophagy‐related genes (ATGs) from HADb and GeneCards was intersected with DEGs to identify differentially expressed ATGs (DEATGs). Functional analyses included Gene Ontology (GO) and KEGG pathway enrichment, protein–protein interaction (PPI) network construction, hub gene identification, immune cell infiltration profiling via single‐sample gene set enrichment analysis (ssGSEA), and molecular docking to evaluate predicted interactions between latanoprost and hub proteins. A total of 990 DEGs were identified, including 15 DEATGs. Among these, S100A8 and S100A9 emerged as hub genes, exhibiting strong functional similarity and central roles within the PPI network. Enrichment analysis revealed significant involvement in autophagy regulation, tyrosine metabolism, and oxidative phosphorylation. Notably, molecular docking predicted high‐affinity binding between latanoprost and S100A9. Immune profiling demonstrated significant alterations in both innate and adaptive immune cell populations, including a strong positive correlation between S100A9 expression and Th2 cell abundance. These findings suggest that S100A9 may act as a central regulator linking autophagy deficiency to immune dysregulation in POAG. Its predicted interaction with latanoprost highlights a potential molecular mechanism for pharmacologic modulation of TM homeostasis, supporting the therapeutic value of targeting S100A9‐mediated autophagy–immune crosstalk in intraocular pressure control.

## 1. Introduction

Primary open‐angle​ glaucoma (POAG) is an optic neuropathy characterized by progressive degeneration of retinal ganglion cells, ultimately leading to irreversible vision loss. As the most prevalent glaucoma subtype globally, POAG accounts for approximately 90% of all cases and remains a major cause of blindness worldwide, yet the precise molecular mechanisms driving disease progression remain incompletely elucidated [1]. Elevated intraocular pressure (IOP), the primary modifiable risk factor, results from impaired aqueous humor (AH) drainage, with the trabecular meshwork (TM) contributing approximately 75% of the total outflow resistance within the conventional pathway [2]. TM dysfunction thus represents a central pathological event that drives IOP elevation in POAG [3]. Current first‐line IOP‐lowering therapies, including β‐blockers and prostaglandin analogs, mainly reduce AH production or enhance uveoscleral (unconventional) outflow. While these interventions offer symptomatic relief, they consistently fail to restore TM cellular homeostasis, which is a core pathological defect, resulting in limited long‐term efficacy [4]. Therefore, uncovering the molecular determinants of TM dysfunction is critical for developing targeted therapeutic strategies, for which transcriptomic analysis of TM tissue remains the gold standard. Autophagy, a conserved intracellular degradation process, maintains cellular homeostasis by eliminating damaged organelles and proteins [5]. Autophagic impairment is involved in numerous human diseases, including neurodegenerative conditions such as Alzheimer’s and Parkinson’s diseases [6], as well as ocular disorders like age‐related macular degeneration and retinitis pigmentosa [7]. Emerging evidence suggests that dysregulated autophagy plays a central role in TM dysfunction. For instance, enhancing the ER–autophagy pathway alleviates nerve injury by degrading mutant myocilin in a myocilin‐related POAG mouse model [8]. TM cells from glaucoma patients also exhibit impaired autophagy and reduced responses to oxidative stress [9]. Concurrently, POAG displays features of immune dysregulation, with infiltration of immune cells such as CD8+ T cells and macrophages contributing to neuroinflammation [10, 11]. Nevertheless, the molecular network linking autophagy to immunopathological mechanisms in POAG remains poorly defined. To address this gap, we employed an integrated bioinformatics approach to analyze transcriptomic datasets from human TM tissues (GSE4316 and GSE27276). Our workflow combined the identification of differentially expressed genes (DEGs) and autophagy‐related genes (ATGs), functional enrichment analyses using Gene Ontology (GO) and Kyoto Encyclopedia of Genes and Genomes (KEGG), protein–protein interaction (PPI) network construction, and regulatory network inference involving microRNAs (miRNAs) and transcription factors (TFs). We also characterized the immune microenvironment using CIBERSORT. Finally, molecular docking simulations were conducted to evaluate predicted interactions between the IOP‐lowering drug latanoprost (CID: 5311221) and identified hub proteins. Among these candidates, S100A9 emerged as a central hub gene linking autophagy dysfunction with type 2 T helper (Th2)–skewed immunopathology (*r* = 0.61). Molecular docking predicted high‐affinity binding between S100A9 and latanoprost (−6.9 kcal/mol, with 36 amino acid interactions). While these results are based on computational modeling, they suggest a plausible mechanism by which latanoprost may modulate S100A9‐related autophagy–immune signaling pathways. These findings provide novel insights into POAG pathogenesis and highlight S100A9 as a potential therapeutic target for restoring TM homeostasis.

## 2. Materials and Methods

### 2.1. Differential Gene Expression Analysis

Transcriptomic datasets GSE4316 (GPL570 platform; 2 POAG samples and 3 control samples) and GSE27276 (GPL2507 platform; 17 POAG samples and 19 control samples) were retrieved from the Gene Expression Omnibus (GEO) using the GEOquery package [12–14]. It is important to note that the GSE4316 cohort has a limited sample size (3 control and 2 POAG samples). Despite this limitation, it was included due to the exceptional rarity of genome‐wide expression data derived from directly microdissected human TM tissue. To mitigate risks associated with a smaller sample size, stringent statistical thresholds (FDR‐adjusted *p* < 0.05 and |log2FC| > 1) were applied for differential expression analysis. Raw expression data underwent batch‐effect correction using the ComBat algorithm, followed by quantile normalization and probe‐to‐gene annotation. Principal component analysis (PCA) was performed to assess data quality and sample clustering. DEGs were identified using the limma package (v3.60.4), with thresholds set at |log2 fold change| > 1 and adjusted *p* value < 0.05. ATGs were collected from the Human Autophagy Database (HADb; https://autophagy.lu) and GeneCards (https://www.genecards.org). After removing duplicate entries, a total of 615 unique ATGs were retained. Differentially expressed autophagy‐related genes (DEATGs) were identified by intersecting the ATG list with the DEGs.

### 2.2. Functional and Pathway Enrichment

Functional similarity among DEATGs was assessed using the GOSemSim package (v2.24.0) based on the Resnik semantic similarity algorithm [15]. GO and KEGG pathway enrichment analyses were conducted using the clusterProfiler package (v4.0.5). Enriched terms and pathways with *p* values < 0.05 were considered statistically significant [16].

### 2.3. Gene Set Enrichment Analysis (GSEA)

GSEA was performed using the c2.cp.v7.2.symbols.gmt gene set from the Molecular Signatures Database (MSigDB v7.5.1). Significantly enriched pathways were identified using clusterProfiler (v4.0.5), with a significance threshold of *p* < 0.05.

### 2.4. PPI Network Analysis

PPI networks were constructed using the STRING database (v11.5), with a minimum interaction confidence score of 0.4. The resulting networks were visualized using Cytoscape (v3.9.1). Key functional modules within the PPI network were identified using the MCODE plugin (v2.0.0). Hub genes were determined using the cytoHubba plugin based on five centrality measures: maximal clique centrality (MCC), maximum neighborhood component (MNC), edge percolated component (EPC), degree, and closeness. The top 15 genes ranked by each algorithm were selected for further analysis.

### 2.5. Immune Infiltration Analysis

The relative abundance of immune cell populations was estimated using single‐sample gene set enrichment analysis (ssGSEA). Enrichment scores for 28 immune cell types were calculated and visualized using boxplots. Correlations between hub gene expression levels and immune cell infiltration were evaluated using Spearman’s rank correlation test. Statistical significance was defined as *p* < 0.05.

### 2.6. Regulatory Network Construction

miRNA–hub gene regulatory networks were predicted using miRTarBase (v9.0) and ENCORI (v3.0). TF–hub gene regulatory relationships were identified using the TRRUST database (v2.0). All regulatory networks were visualized using Cytoscape.

### 2.7. Molecular Docking

Molecular docking analysis was performed using CB‐Dock2. The three‐dimensional structure of latanoprost (CID: 5311221) was obtained from PubChem. Protein structures of hub genes were retrieved from the Protein Data Bank, including HP (PDB ID: 5HU6), LCN2 (1L6M), S100A9 (4GGF), S100A12 (1ODB), S100A8 (4XJK), and KRT5 (6JFV). Blind docking was conducted using the AutoDock Vina algorithm. Docking poses were visualized and analyzed to assess predicted binding conformations. Binding affinity was evaluated based on the Vina score and the number of amino acid residues interacting with latanoprost.

### 2.8. Statistical Analysis

All statistical analyses were performed using R software (v4.0.2). Continuous variables were analyzed using Student’s *t*‐test for normally distributed data and the Mann–Whitney U test for nonnormally distributed data. Statistical significance was defined as a two‐tailed *p* value < 0.05.

## 3. Results

### 3.1. Screening of DEGs

**FIGURE 1:**
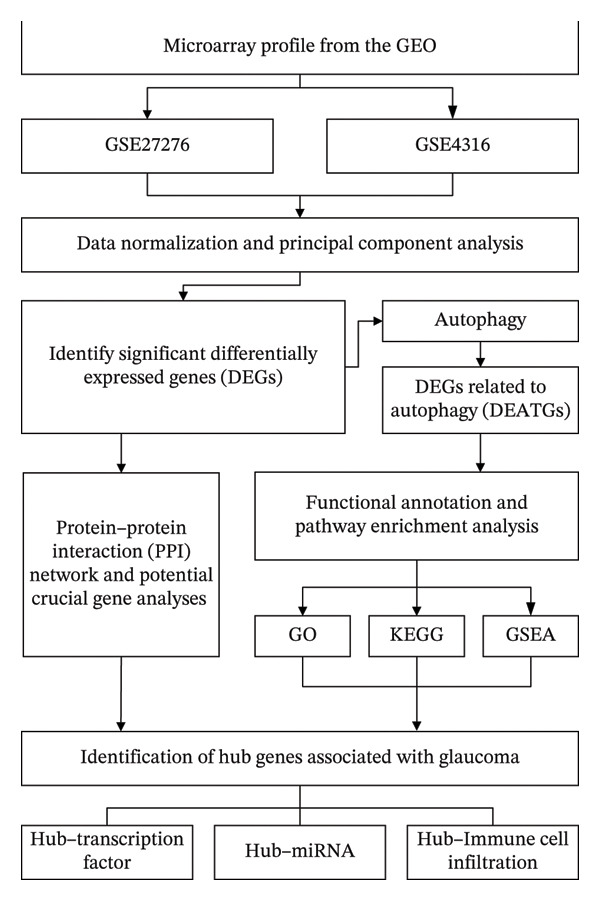
Flowchart of the comprehensive bioinformatics analysis performed in this study to identify autophagy‐related molecular mechanisms in POAG. Abbreviations: DEGs, differentially expressed genes; DEATGs, differentially expressed autophagy‐related genes; POAG, primary open‐angle glaucoma.

The overall workflow of this study is outlined in Figure 1. PCA demonstrated distinct separation between POAG and control samples, indicating reliable sample classification (Figure 2(a)). After quantile normalization (Figures 2(d), 2(e), 2(f), and 2(g)), differential expression analysis identified 176 DEGs in the GSE27276 dataset (64 upregulated and 112 downregulated) and 814 DEGs in GSE4316 (356 upregulated and 458 downregulated), as visualized in volcano plots and heatmaps (Figures 2(b), 2(c), 2(h), and 2(i) and Table 1 **)**. These results confirm reproducible expression changes in POAG‐associated TM tissue.

FIGURE 2Differential gene expression analysis between POAG and control trabecular meshwork tissues. (a) Principal component analysis (PCA) plot. (b, c) Volcano plots of DEGs from (b) GSE27276 and (c) GSE4316 datasets. (d, e) Boxplots of expression distributions before normalization for (d) GSE27276 and (e) GSE4316. (f, g) Boxplots after normalization for (f) GSE27276 and (g) GSE4316. (h, i) Heatmaps of DEGs from (h) GSE27276 and (i) GSE4316.

**Figure:**
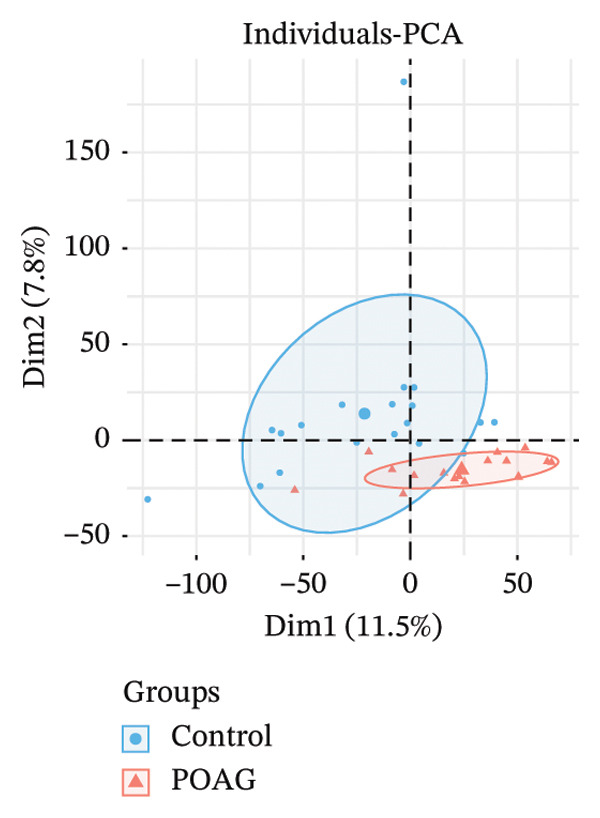
(a)

**Figure:**
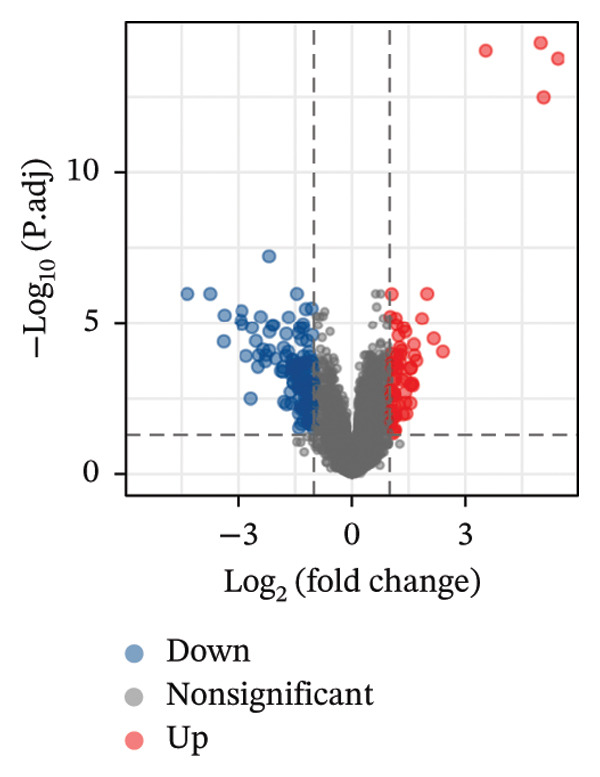
(b)

**Figure:**
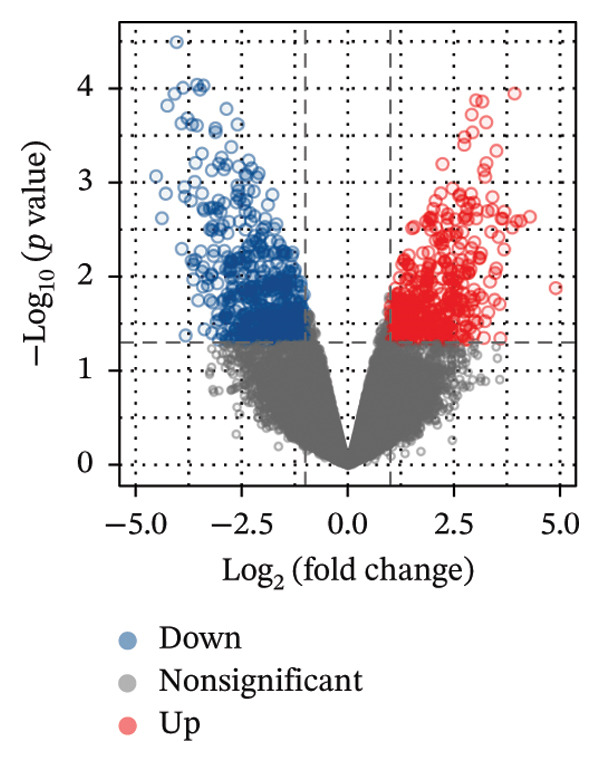
(c)

**Figure:**
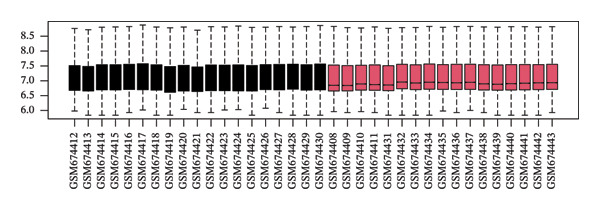
(d)

**Figure:**
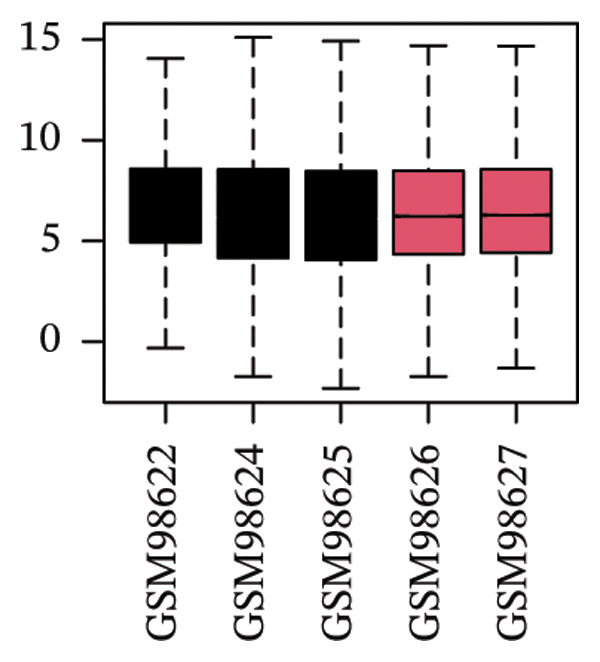
(e)

**Figure:**
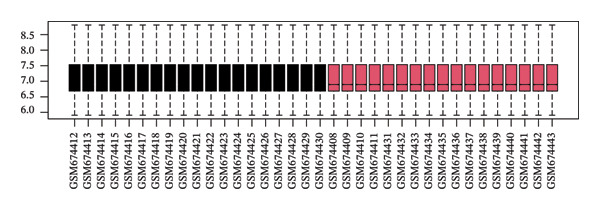
(f)

**Figure:**
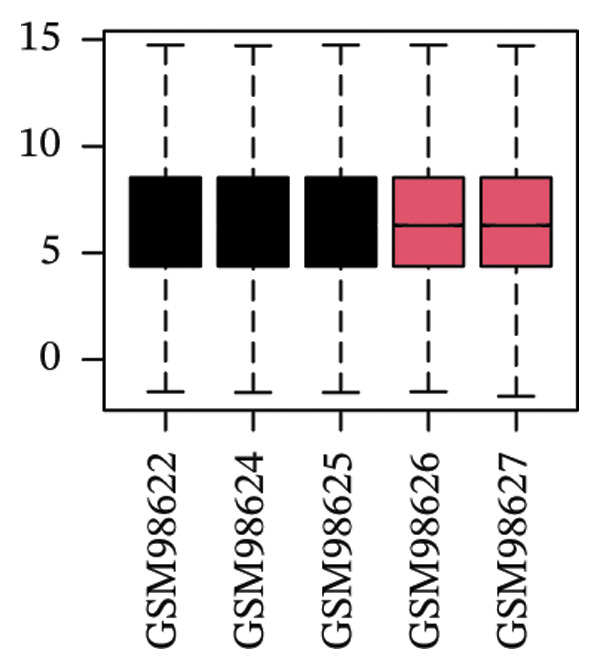
(g)

**Figure:**
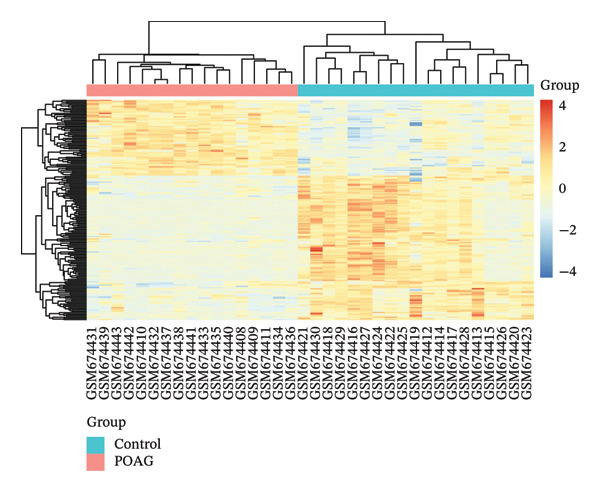
(h)

**Figure:**
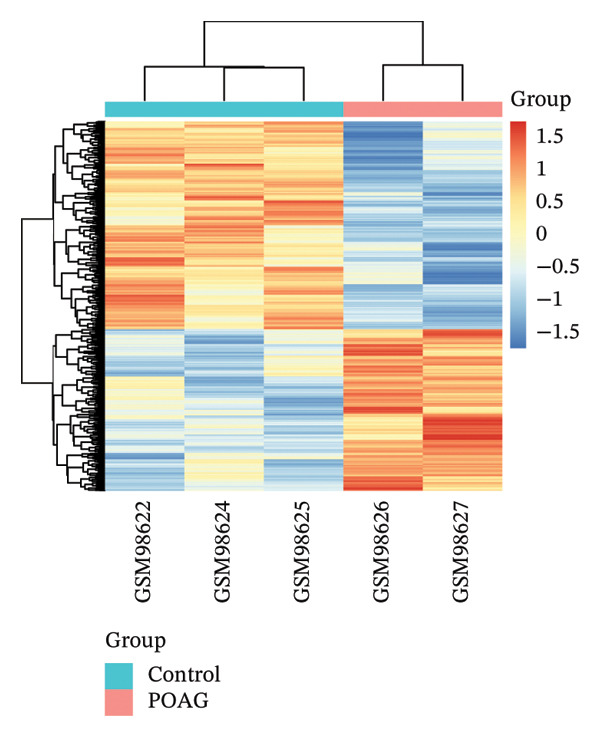
(i)

**TABLE 1:** Data information data sheet.

ID	GEO number	Platforms	Sample	Source types	references
1	GSE4316	GPL570	3 control and 2 patients	Trabecular meshwork tissue	[12]
2	GSE27276	GPL2507	19 control and 17 patients	Trabecular meshwork tissue	[13].

### 3.2. Identification of DEATGs

To identify autophagy‐related components of POAG pathology, DEGs were intersected with a curated set of 615 ATGs, yielding 15 DEATGs (Figures 3(a), 3(b), 3(c), and 3(d)**)**. Chromosomal mapping showed that several DEATGs were spatially clustered, suggesting shared regulatory mechanisms (Figure 4(b)). Functional similarity analysis highlighted S100A8 and S100A9 as closely related genes, consistent with their known heterodimeric activity (Figure 4(a)). PPI network analysis revealed PPARG as a key upstream autophagy regulator among DEATGs (Figure 4(c)), supporting its potential role in TM dysfunction.

FIGURE 3Identification and expression of differentially expressed autophagy‐related genes (DEATGs). (a, b) Boxplots showing DEATG expression levels in (a) GSE4316 and (b) GSE27276 datasets. (c, d) Heatmaps of DEATGs in (c) GSE4316 and (d) GSE27276.

**Figure:**
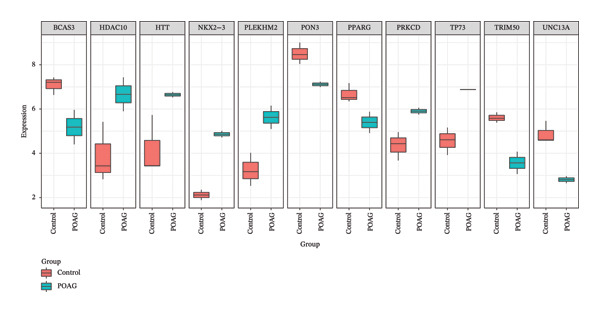
(a)

**Figure:**
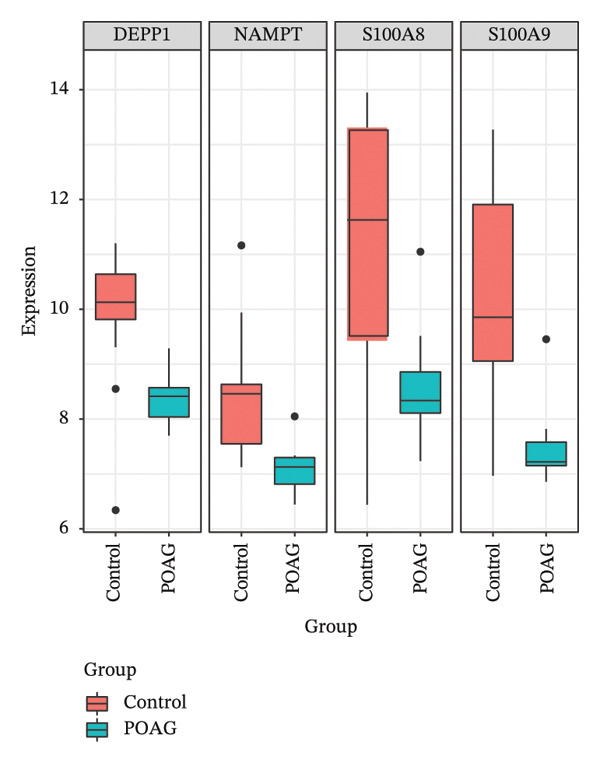
(b)

**Figure:**
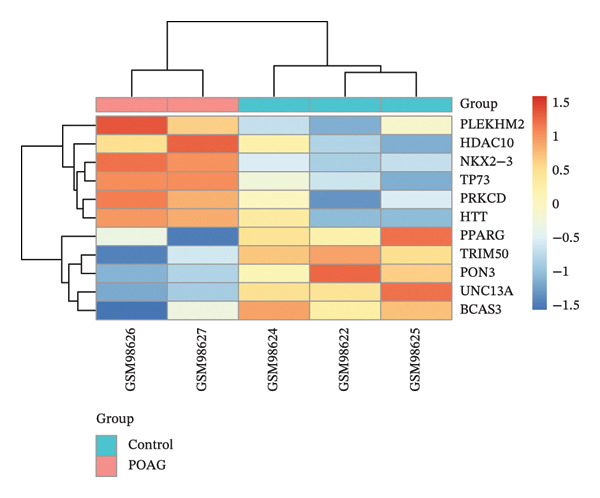
(c)

**Figure:**
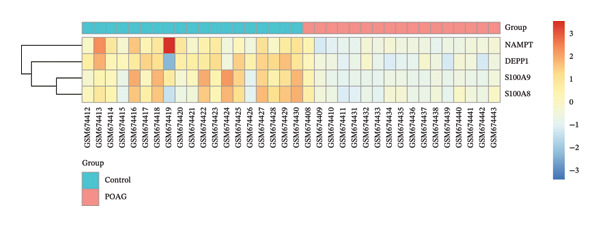
(d)

FIGURE 4Functional characterization of DEATGs. (a) Functional association network (Friends analysis). (b) Chromosomal locations of DEATGs. (c) Protein–protein interaction (PPI) network of DEATGs, with node darkness indicating maximal clique centrality (MCC) scores.

**Figure:**
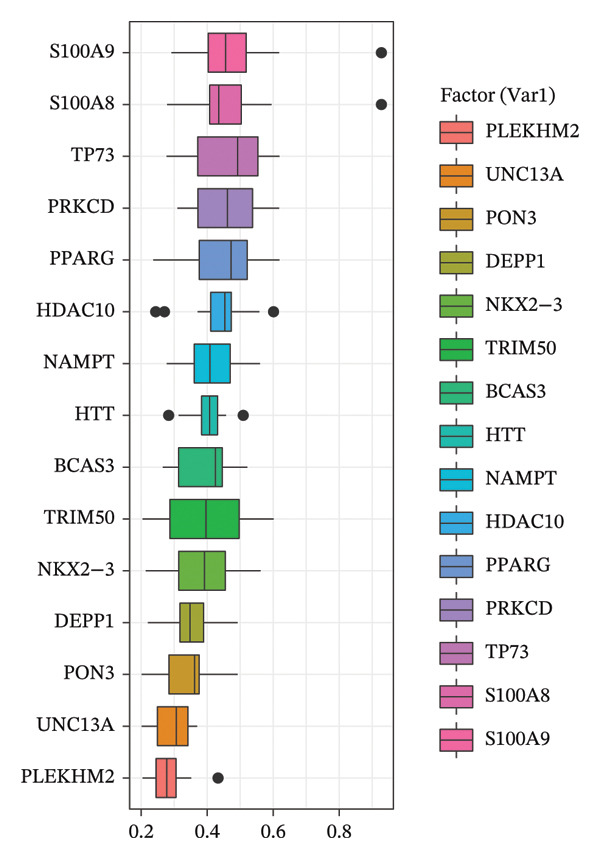
(a)

**Figure:**
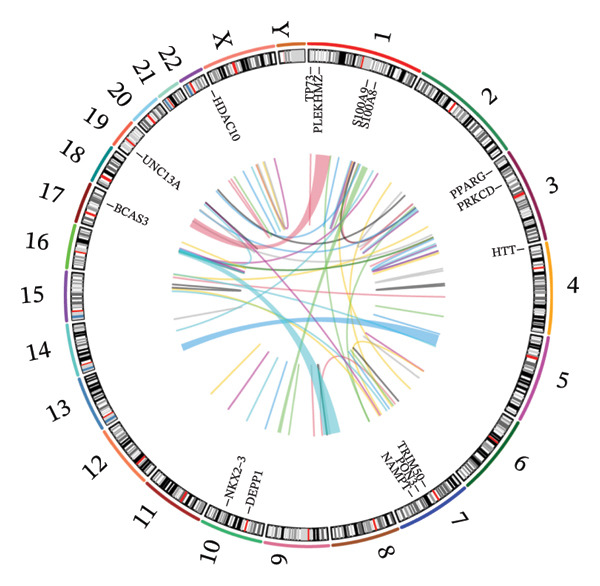
(b)

**Figure:**
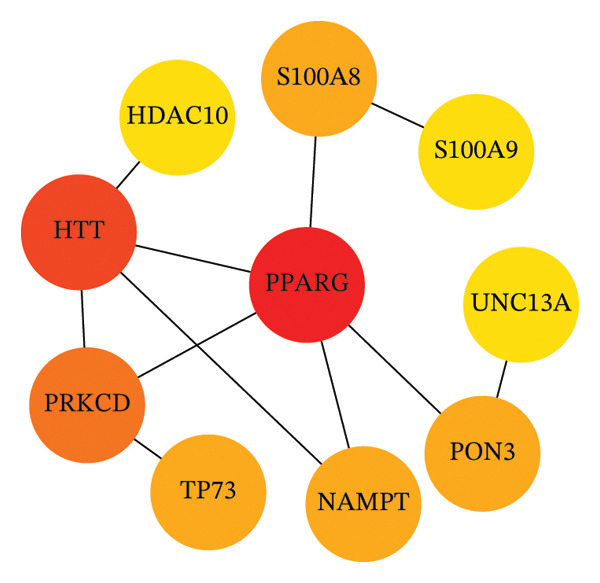
(c)

### 3.3. Functional Enrichment Analysis

GO analysis of DEATGs revealed enrichment in processes central to autophagic and apoptotic regulation. Specifically, biological processes (BPs) were enriched for autophagy and modulation of apoptotic signaling pathways. Cellular components (CCs) were enriched in vesicle‐associated compartments, including the secretory granule lumen and cytoplasmic vesicles. Molecular functions (MFs) were enriched for lipid‐binding activity, particularly fatty acid and monocarboxylic acid binding (Figures 5(a), 5(b), 5(c), 5(d), 5(e), and 5(f) and Table 2). KEGG pathway enrichment analysis of all DEGs highlighted involvement in tyrosine metabolism (hsa00350) and immune‐related pathways such as *Staphylococcus aureus* infection (hsa05150), suggesting that both metabolic and inflammatory mechanisms are active in the glaucomatous TM (Figures 5(g) and 5(h) and Table 3).

FIGURE 5Gene ontology (GO) and Kyoto Encyclopedia of Genes and Genomes (KEGG) enrichment analyses. (a) GO term–gene interaction network. (b) Bubble plot of top enriched GO terms in biological process (BP), cellular component (CC), and molecular function (MF) categories. (c–e) Chord diagrams showing DEATG–GO term associations for (c) BP, (D) CC, and (e) MF. (f) Circular visualization of GO analysis results. (g) Chord diagram of KEGG pathway–gene interactions. (h) Bubble chart of KEGG pathway enrichment.

**Figure:**
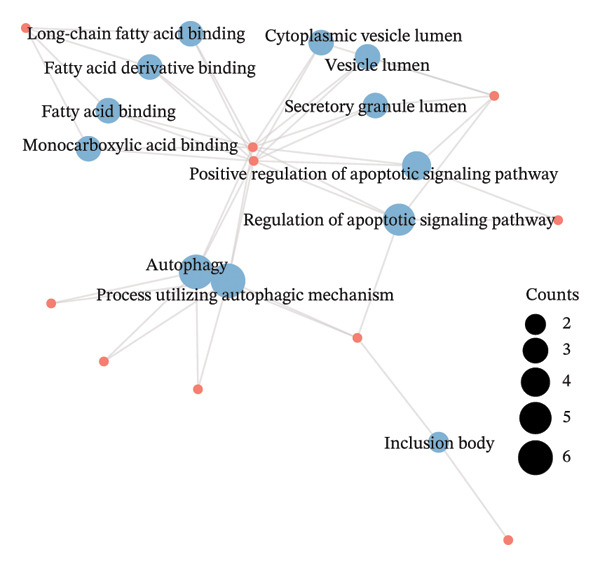
(a)

**Figure:**
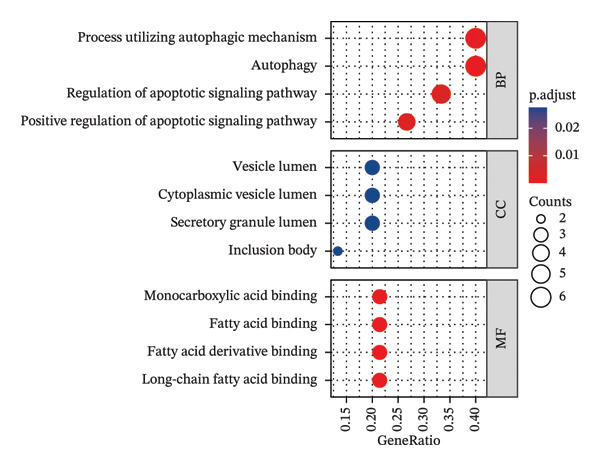
(b)

**Figure:**
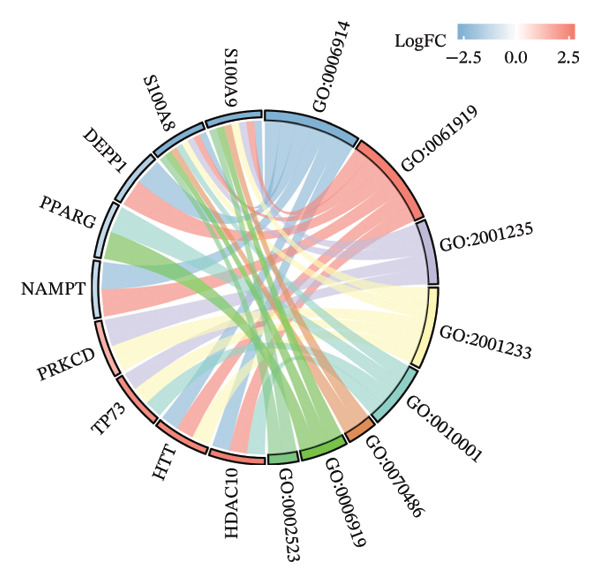
(c)

**Figure:**
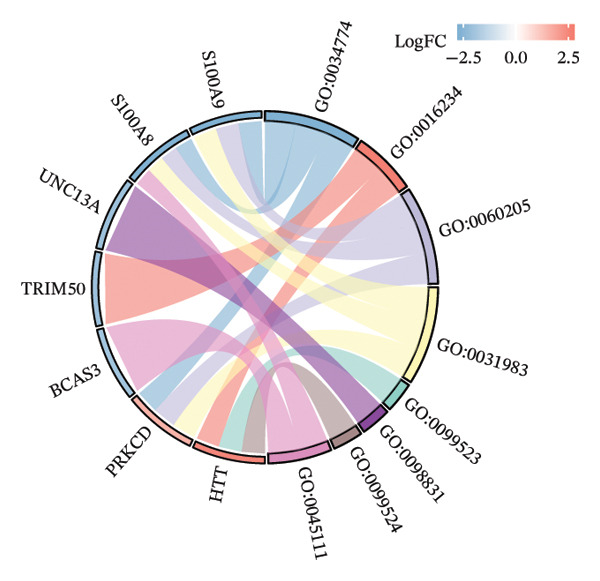
(d)

**Figure:**
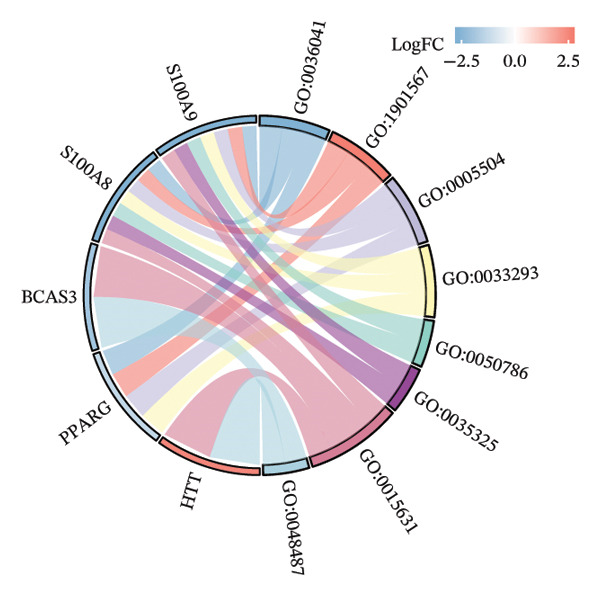
(e)

**Figure:**
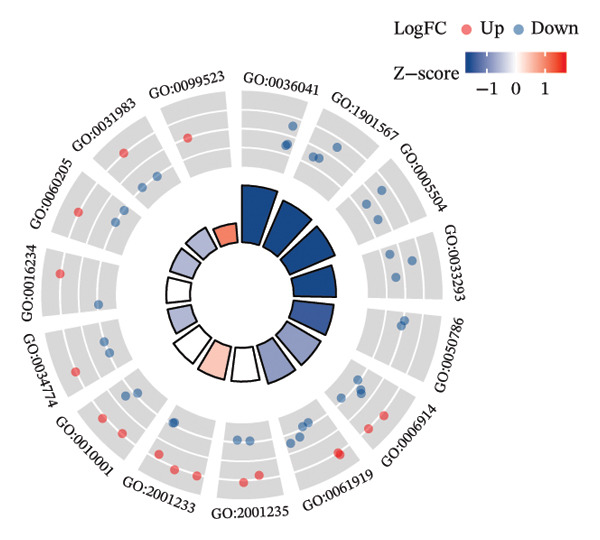
(f)

**Figure:**
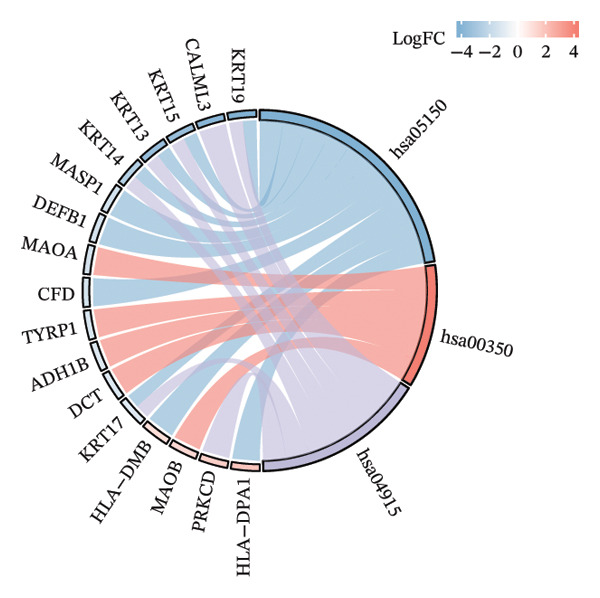
(g)

**Figure:**
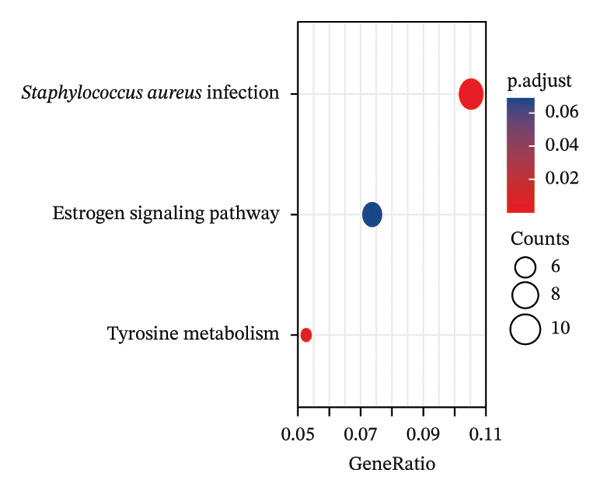
(h)

**TABLE 2:** GO enrichment analysis of differentially expressed genes.

Ontology	ID	Description	GeneRatio	BgRatio	*p* value	*p* adjust	q value
BP	GO:0006914	Autophagy	6/15	496/18670	1.39e‐06	6.45e‐04	3.64e‐04
BP	GO:0061919	Process utilizing the autophagic mechanism	6/15	496/18670	1.39e‐06	6.45e‐04	3.64e‐04
BP	GO:2001235	Positive regulation of the apoptotic signaling pathway	4/15	179/18670	1.03e‐05	0.003	0.002
BP	GO:2001233	Regulation of the apoptotic signaling pathway	5/15	406/18670	1.19e‐05	0.003	0.002
CC	GO:0034774	Secretory granule lumen	3/15	321/19717	0.002	0.028	0.021
CC	GO:0016234	Inclusion body	2/15	82/19717	0.002	0.028	0.021
CC	GO:0060205	Cytoplasmic vesicle lumen	3/15	338/19717	0.002	0.028	0.021
CC	GO:0031983	Vesicle lumen	3/15	339/19717	0.002	0.028	0.021
MF	GO:0036041	Long‐chain fatty acid binding	3/14	14/17697	1.43e‐07	1.06e‐05	4.81e‐06
MF	GO:1901567	Fatty acid derivative binding	3/14	28/17697	1.28e‐06	4.72e‐05	2.15e‐05
MF	GO:0005504	Fatty acid binding	3/14	34/17697	2.32e‐06	5.73e‐05	2.61e‐05
MF	GO:0033293	Monocarboxylic acid binding	3/14	64/17697	1.60e‐05	2.95e‐04	1.34e‐04

**TABLE 3:** KEGG enrichment analysis of differentially expressed genes.

Ontology	ID	Description	GeneRatio	BgRatio	*p* value	*p* adjust	q value
KEGG	hsa05150	*Staphylococcus aureus* infection	10/95	96/8076	1.54e‐07	2.75e‐05	2.69e‐05
KEGG	hsa00350	Tyrosine metabolism	5/95	36/8076	5.72e‐05	0.005	0.005
KEGG	hsa04915	Estrogen signaling pathway	7/95	138/8076	0.001	0.069	0.067

### 3.4. GSEA

GSEA further clarified the biological landscape associated with POAG. Enriched gene sets included mitochondrial electron transport chain components, pathways related to metabolic diseases, and extracellular matrix (ECM) proteoglycans, pointing to impaired energy metabolism and ECM remodeling in TM cells. Additional enrichment was observed in the vitamin D receptor (VDR) pathway, p53‐mediated stress response, interleukin signaling, and ERK inactivation (Figures 6(a), 6(b), 6(c), 6(d), 6(e), 6(f), 6(g), and 6(h)). These findings support a model in which autophagy dysfunction contributes to oxidative stress, altered immune signaling, and fibrotic changes in the TM.

FIGURE 6Gene set enrichment analysis (GSEA) of DEGs. (a) Overview of five major biological processes significantly enriched in POAG. (b–h) Detailed enrichment plots: (b) electron transport chain/oxidative phosphorylation system in mitochondria, (c) metabolic diseases, (d) ECM proteoglycans, (e) vitamin D receptor pathway, (f) p53 downstream pathway, (g) interleukin signaling, and (h) ERK inactivation.

**Figure:**
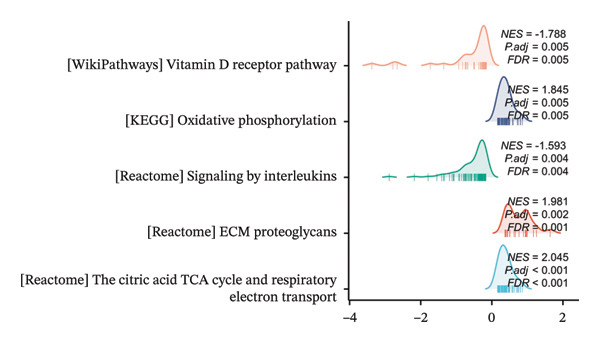
(a)

**Figure:**
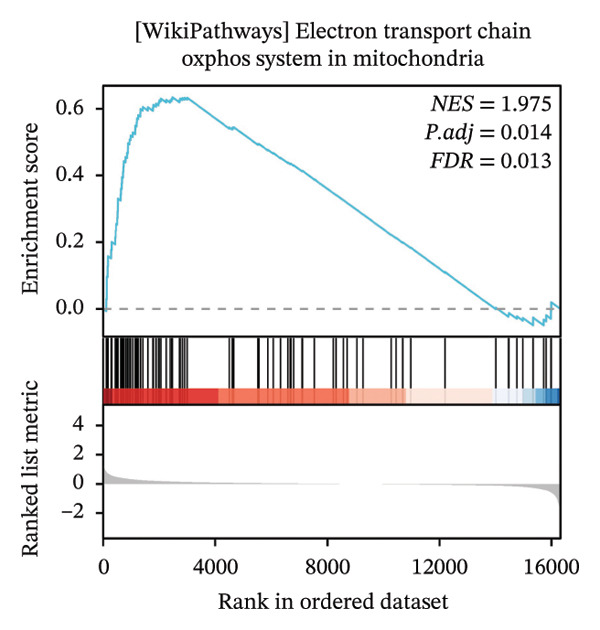
(b)

**Figure:**
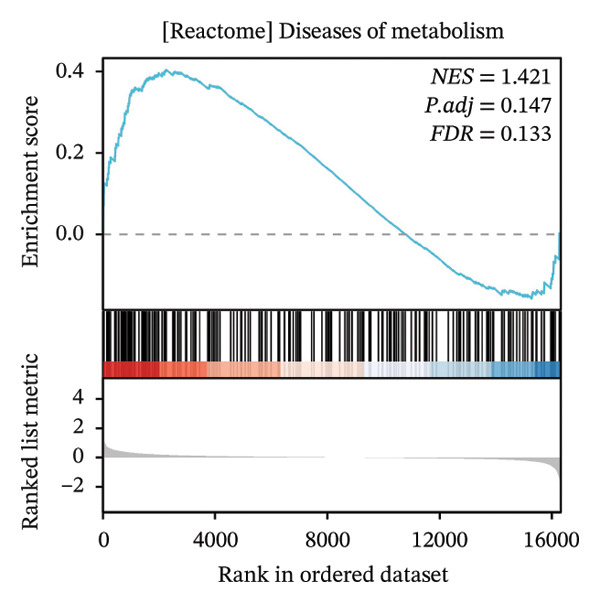
(c)

**Figure:**
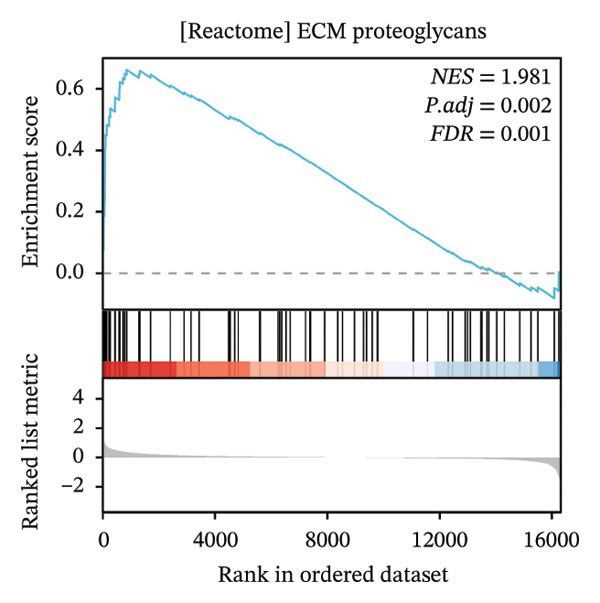
(d)

**Figure:**
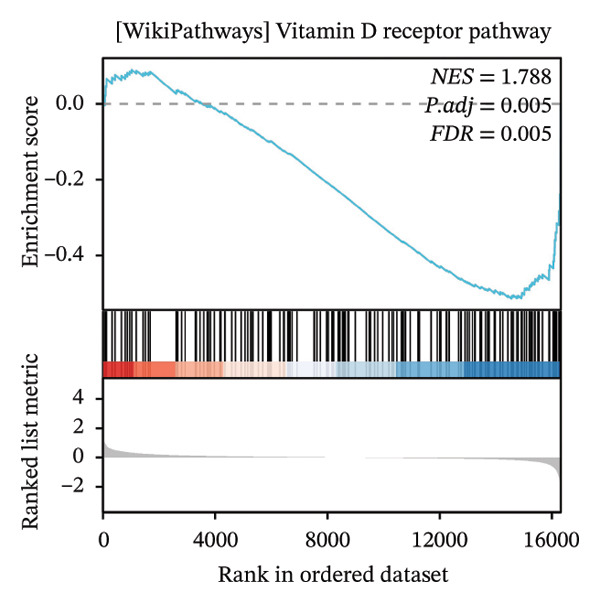
(e)

**Figure:**
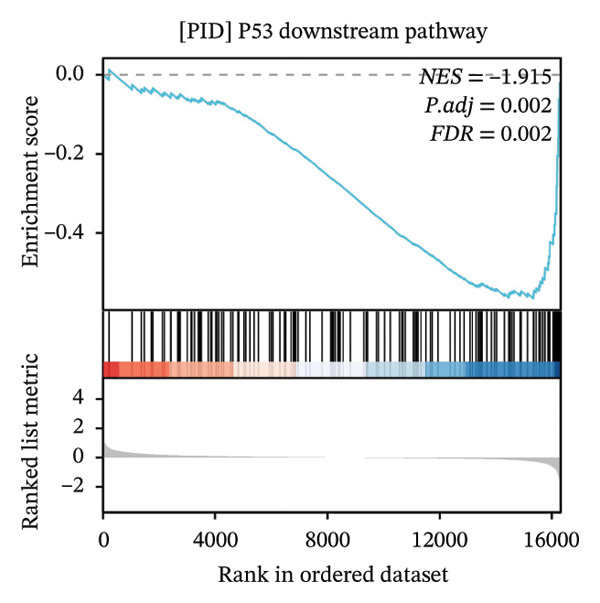
(f)

**Figure:**
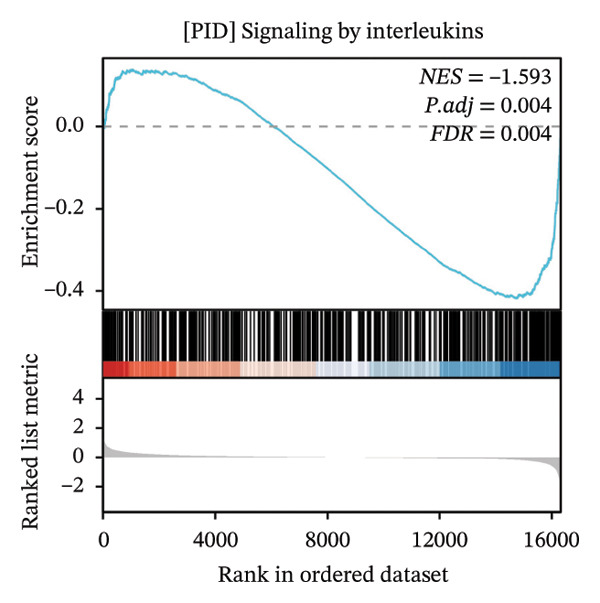
(g)

**Figure:**
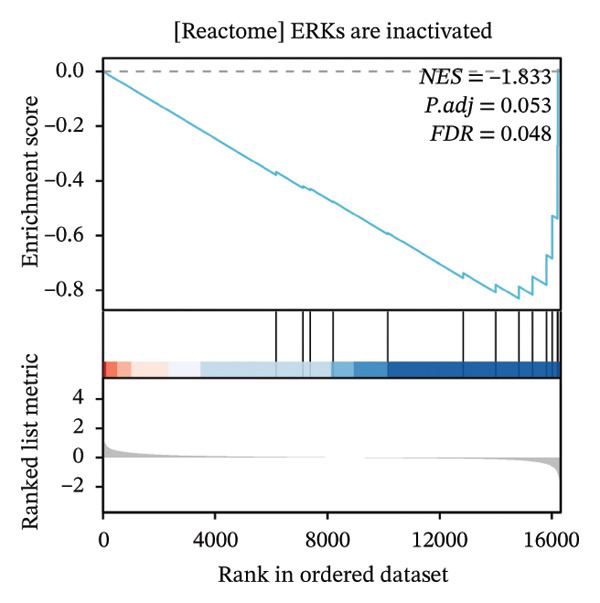
(h)

### 3.5. Hub Gene Identification

The PPI network constructed from DEGs consisted of 138 nodes and 282 edges (Figures 7(a) and 7(c)). Top‐ranked hub genes were identified using five centrality algorithms and included HP, LCN2, S100A9, S100A12, S100A8, and KRT5 (Figure 7(b) and Table 4 **)**. These genes were prioritized for further regulatory and functional analysis due to their central roles in network topology and relevance to autophagy and immune regulation.

FIGURE 7PPI network construction and hub gene identification. (a) Global PPI network of DEGs. (b) Hub genes identified by five algorithmic methods. (c) PPI network with expression overlay: red indicates upregulation; blue indicates downregulation.

**Figure:**
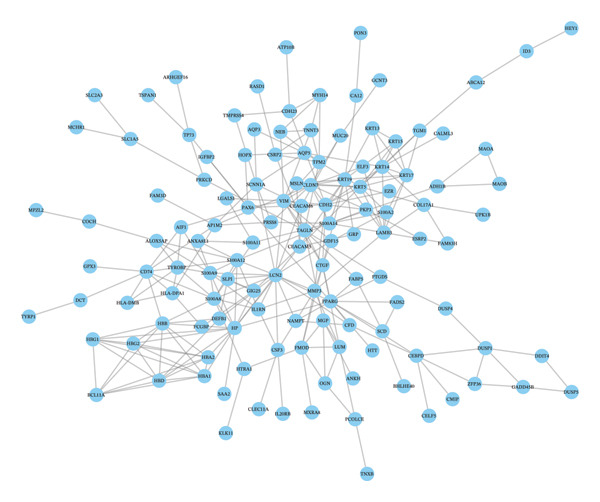
(a)

**Figure:**
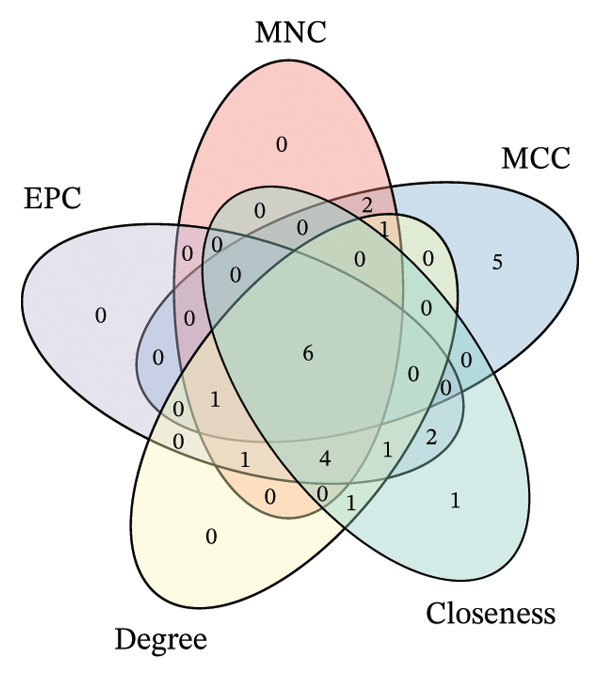
(b)

**Figure:**
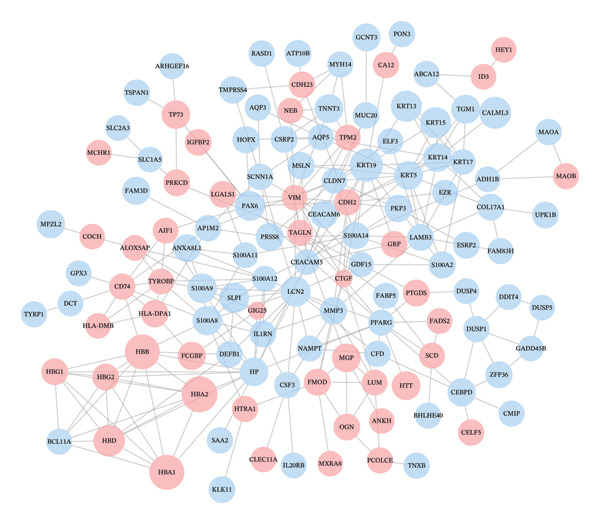
(c)

**TABLE 4:** Analysis of the hub gene in the PPI network.

MCC	MNC	EPC	Degree	Closeness	Intersection set
HBB	HP	LCN2	LCN2	LCN2	HP
HBD	LCN2	HP	HP	HP	LCN2
HBA1	KRT5	CDH2	KRT5	VIM	S100A9
HBA2	S100A9	KRT19	KRT19	PPARG	S100A12
HBG2	VIM	KRT5	PPARG	CDH2	S100A8
HBG1	HBB	S100A8	CDH2	CTGF	KRT5
HP	CDH2	S100A9	VIM	KRT19	/
BCL11A	KRT14	CLDN7	HBB	CLDN7	/
LCN2	S100A8	S100A12	S100A9	MMP3	/
S100A9	CLDN7	VIM	S100A8	S100A12	/
S100A12	S100A12	CTGF	KRT14	S100A8	/
S100A8	CTGF	LAMB3	LAMB3	KRT5	/
KRT5	LAMB3	S100A14	CLDN7	PAX6	/
KRT17	KRT17	MMP3	S100A12	S100A9	/
KRT14	HBD	HBB	CTGF	S100A14	/

### 3.6. Regulatory Networks of Hub Genes

Regulatory network analysis revealed extensive control of hub genes by noncoding RNAs and TFs. For example, KRT5 was targeted by 48 miRNAs, HP by 18, and S100A9 by 8 (Figure 8(a)). TF analysis identified regulatory control by 3‐4 TFs per gene. Specifically, HP was regulated by four TFs, while KRT5, LCN2, and S100A9 were each regulated by three (Figure 8(b)). These regulatory maps underscore the complex transcriptional and posttranscriptional control of key autophagy–immune genes in POAG.

FIGURE 8Regulatory network analyses. (a) miRNA–hub gene interaction network. Pink nodes: hub genes; blue nodes: miRNAs. (b) Transcription factor (TF)–hub gene interaction network. Red nodes: hub genes; green nodes: TFs.

**Figure:**
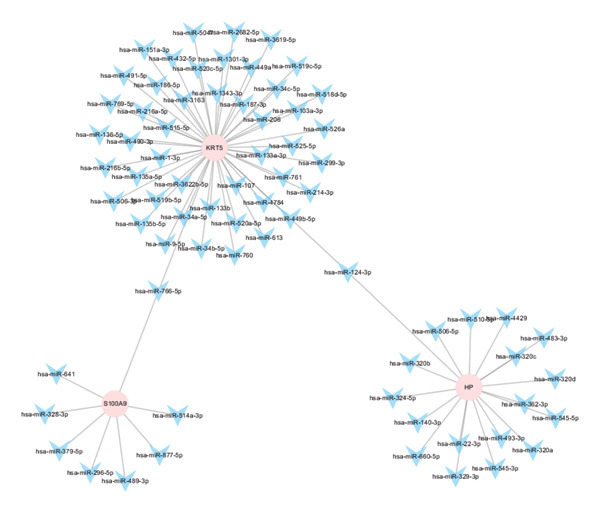
(a)

**Figure:**
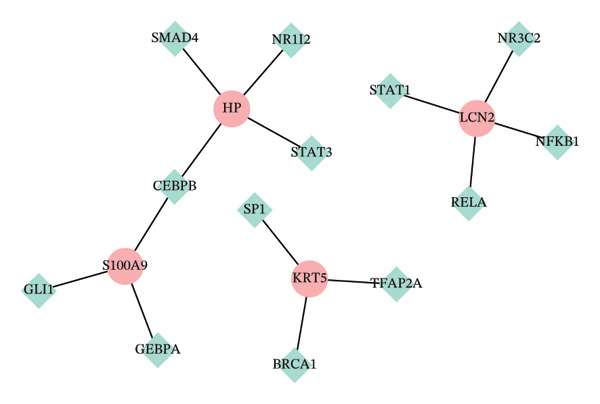
(b)

### 3.7. Immune Microenvironment

Immune infiltration analysis using ssGSEA revealed significant alterations in the TM immune landscape of POAG patients compared to controls. Specifically, POAG samples exhibited increased infiltration of activated CD8+ T cells (*p* < 0.05), macrophages (*p* < 0.001), monocytes (*p* < 0.05), natural killer T (NKT) cells (*p* < 0.001), plasmacytoid dendritic cells (pDCs) (*p* < 0.001), and regulatory T cells (Tregs) (*p* < 0.0001). In contrast, decreased levels were observed in central memory CD8+ T cells (*p* < 0.05), gamma delta T cells (*p* < 0.05), neutrophils (*p* < 0.01), and Th2 cells (*p* < 0.01) (Figures 9(a) and 9(b)).

FIGURE 9Immune cell infiltration analysis in POAG. (a) Proportional abundance of immune cell types in each sample. (b) Comparative analysis of immune cell infiltration between control and POAG samples. ^∗^ *p* < 0.05; ^∗∗^ *p* < 0.01; ^∗∗∗^ *p* < 0.001; ^∗∗∗∗^ *p* < 0.0001.

**Figure:**
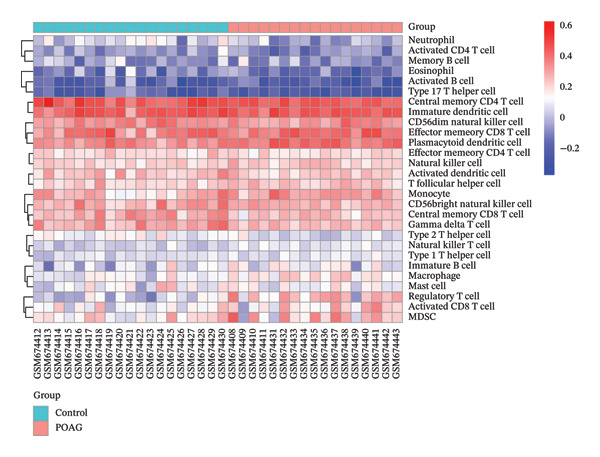
(a)

**Figure:**
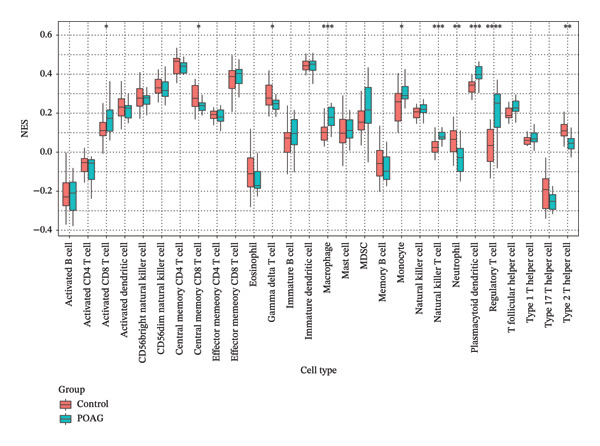
(b)

Correlation analysis further revealed significant associations between hub gene expression and specific immune populations. S100A8 expression positively correlated with CD56+ natural killer (NK) cells (*r* = 0.54) and activated dendritic cells (*r* = 0.56). S100A9 was positively associated with activated CD4+ T cells (*r* = 0.50), CD56dim NK cells (*r* = 0.51), activated dendritic cells (*r* = 0.51), and Th2 cells (*r* = 0.61). KRT5 showed both positive correlations with immature B cells (*r* = 0.51) and macrophages (*r* = 0.57) and a negative correlation with neutrophils (*r* = −0.65) (Figures 10(a), 10(b), 10(c), 10(d), 10(e), 10(f), 10(g), 10(h), and 10(i)). These findings position S100A9 and S100A8 as key immunomodulatory molecules potentially acting through autophagy–immune crosstalk.

FIGURE 10Correlation analysis between hub genes and immune cells. (a, b) S100A8 expression shows positive correlations with (a) CD56dim natural killer cells (*r* = 0.54) and (b) activated dendritic cells (*r* = 0.56). (c–f) S100A9 expression positively correlates with (c) activated CD4 T cells (*r* = 0.50), (d) CD56dim natural killer cells (*r* = 0.51), (e) activated dendritic cells (*r* = 0.51), and (f) type 2 T helper cells (*r* = 0.61). (g–i) KRT5 expression shows positive correlations with (g) immature B cells (*r* = 0.51) and (h) macrophages (*r* = 0.57) and negative correlation with (i) neutrophils (*r* = −0.65). All correlations are significant at *p* < 0.05.

**Figure:**
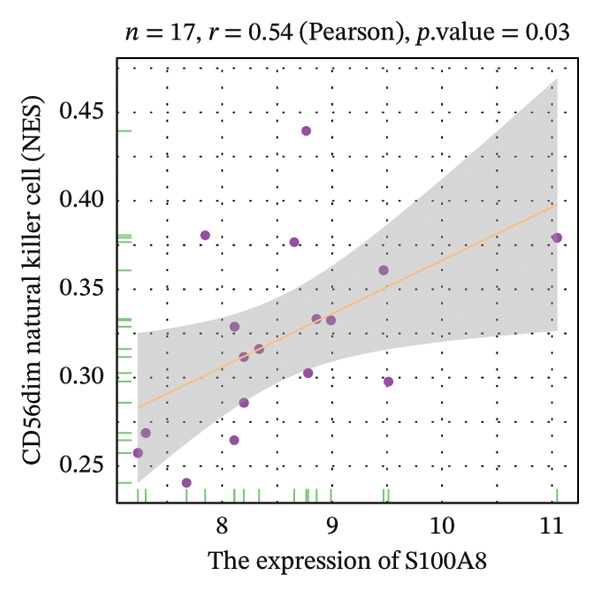
(a)

**Figure:**
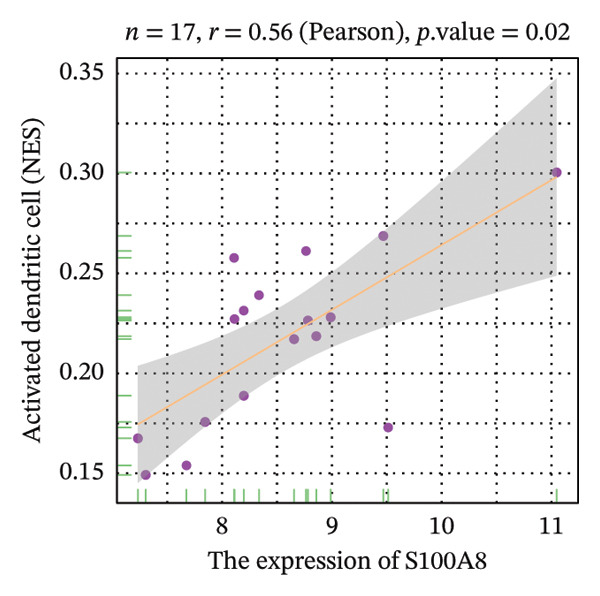
(b)

**Figure:**
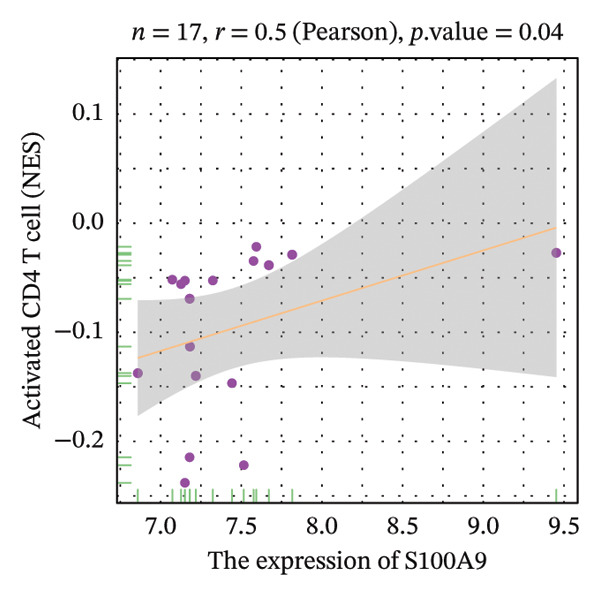
(c)

**Figure:**
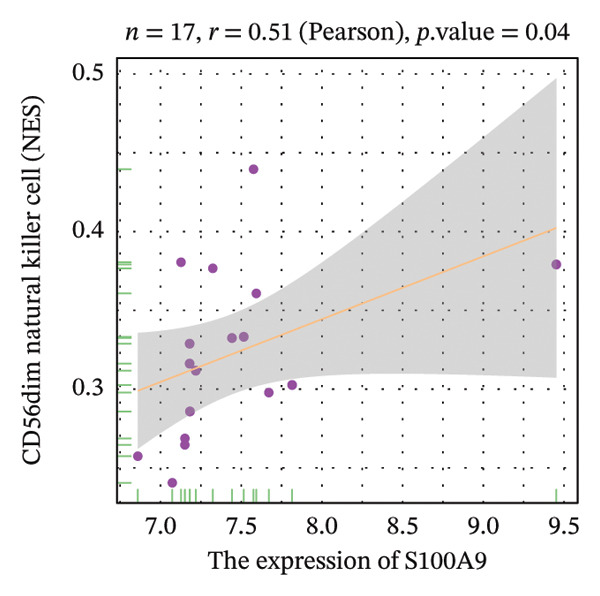
(d)

**Figure:**
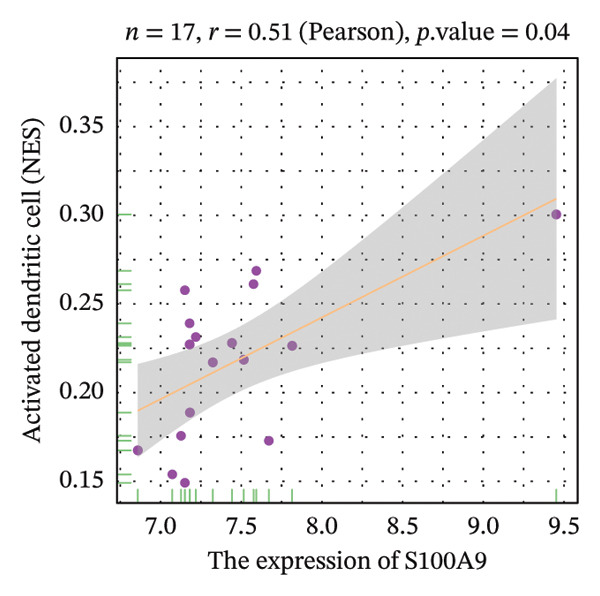
(e)

**Figure:**
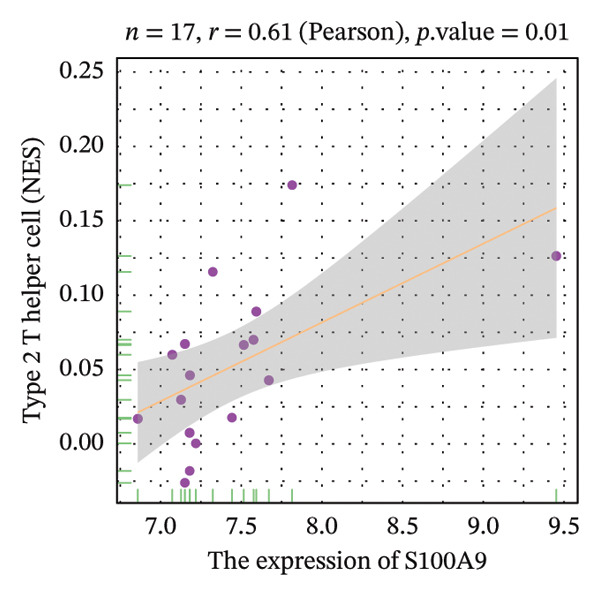
(f)

**Figure:**
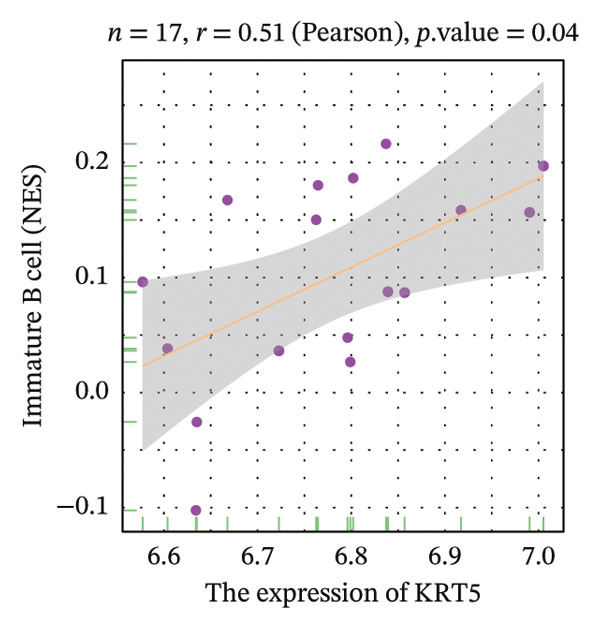
(g)

**Figure:**
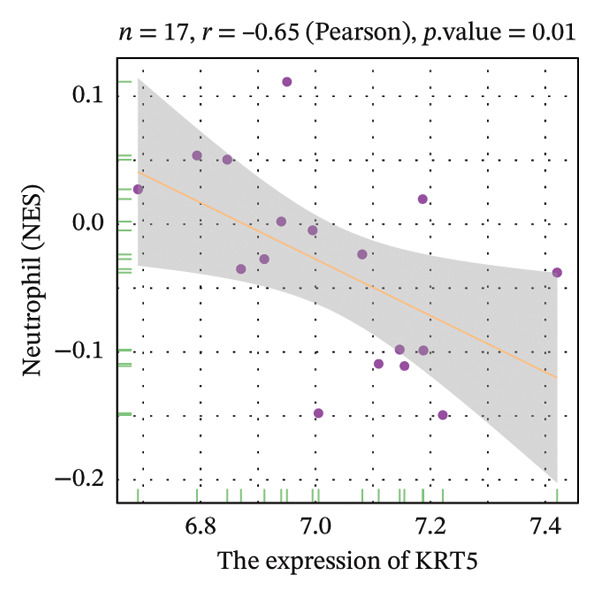
(h)

**Figure:**
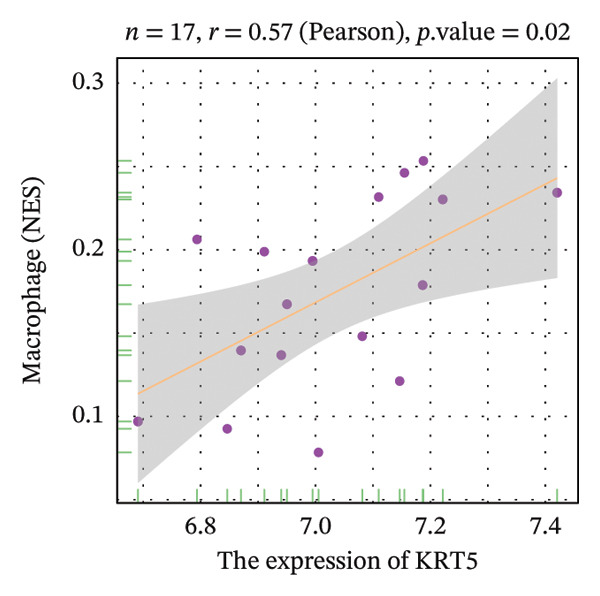
(i)

### 3.8. Molecular Docking Analysis

To assess potential pharmacologic modulation, molecular docking simulations were performed between latanoprost and hub proteins. S100A9 exhibited a strong predicted binding affinity to latanoprost, with a Vina score of −6.9 kcal/mol and 36 amino acid interactions, indicating a stable docking conformation. Similarly, HP showed a Vina score of −7.3 kcal/mol with 28 interactions (Figures 11(a) and 11(c) and Table 5). Additional interactions were observed with LCN2 (−7.3 kcal/mol), S100A12 (−6.2 kcal/mol), S100A8 (−5.9 kcal/mol), and KRT5 (−5.3 kcal/mol), primarily through hydrophobic forces (Figures 12(b), 12(a), and 12(c)). Notably, high‐affinity binding conformations involved more than 15 atomic contacts within deep protein binding pockets. These findings suggest that latanoprost may modulate autophagy–immune signaling in POAG through its predicted interaction with S100A9, although functional validation is required.

FIGURE 11Molecular docking analysis of latanoprost with hub proteins. (a) Strong binding affinity to HP (−7.3 kcal/mol; 28 interacting amino acids). (b) Moderate binding to LCN2 (−7.3 kcal/mol). (c) Moderate binding to S100A9 (−6.9 kcal/mol; 36 interacting amino acids) via hydrogen bonding, ionic interactions, and π‐stacking.

**Figure:**
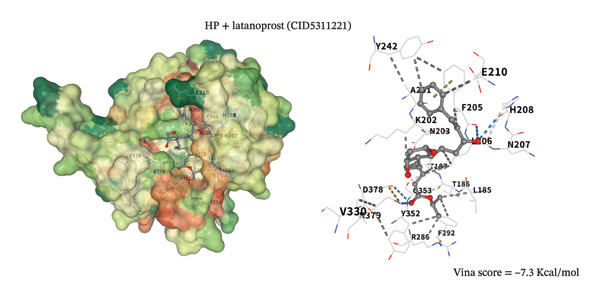
(a)

**Figure:**
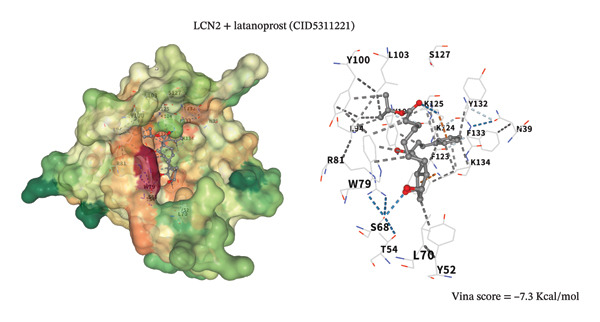
(b)

**Figure:**
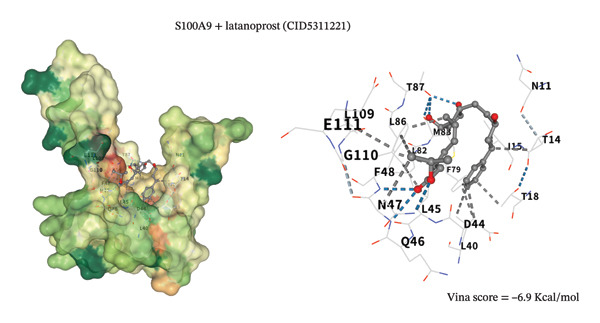
(c)

**TABLE 5:** Components and targets.

Component name	PubChem CID	Targets
Propan‐2‐yl (Z)‐7‐[(1R,2R,3R,5S)‐3,5‐dihydroxy‐2‐[(3R)‐3‐hydroxy‐5‐phenylpentyl]cyclopentyl]hept‐5‐enoate	5311221	HP
Propan‐2‐yl (Z)‐7‐[(1R,2R,3R,5S)‐3,5‐dihydroxy‐2‐[(3R)‐3‐hydroxy‐5‐phenylpentyl]cyclopentyl]hept‐5‐enoate	5311221	LCN2
Propan‐2‐yl (Z)‐7‐[(1R,2R,3R,5S)‐3,5‐dihydroxy‐2‐[(3R)‐3‐hydroxy‐5‐phenylpentyl]cyclopentyl]hept‐5‐enoate	5311221	S100A9
Propan‐2‐yl (Z)‐7‐[(1R,2R,3R,5S)‐3,5‐dihydroxy‐2‐[(3R)‐3‐hydroxy‐5‐phenylpentyl]cyclopentyl]hept‐5‐enoate	5311221	S100A12
Propan‐2‐yl (Z)‐7‐[(1R,2R,3R,5S)‐3,5‐dihydroxy‐2‐[(3R)‐3‐hydroxy‐5‐phenylpentyl]cyclopentyl]hept‐5‐enoate	5311221	S100A8
Propan‐2‐yl (Z)‐7‐[(1R,2R,3R,5S)‐3,5‐dihydroxy‐2‐[(3R)‐3‐hydroxy‐5‐phenylpentyl]cyclopentyl]hept‐5‐enoate	5311221	KRT5

FIGURE 12Molecular docking analysis of latanoprost with additional hub proteins. (a) S100A12 (−6.2 kcal/mol), (b) S100A8 (−5.9 kcal/mol), and (c) KRT5 (−5.3 kcal/mol), primarily mediated by hydrophobic interactions.

**Figure:**
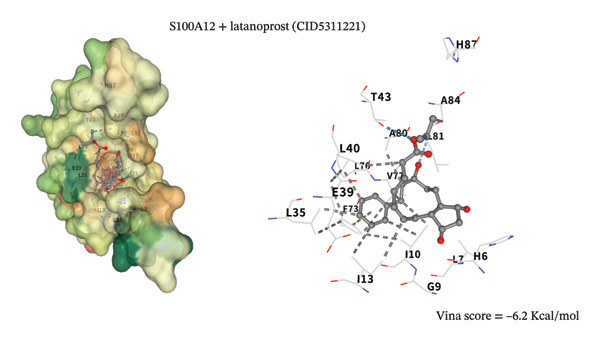
(a)

**Figure:**
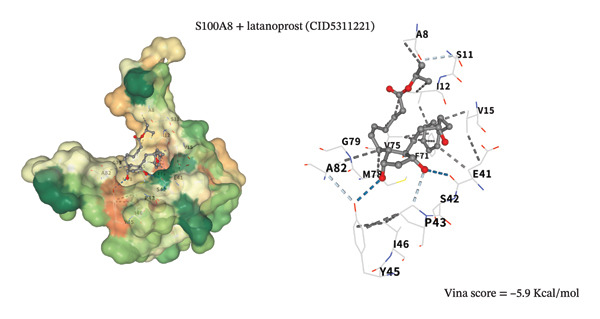
(b)

**Figure:**
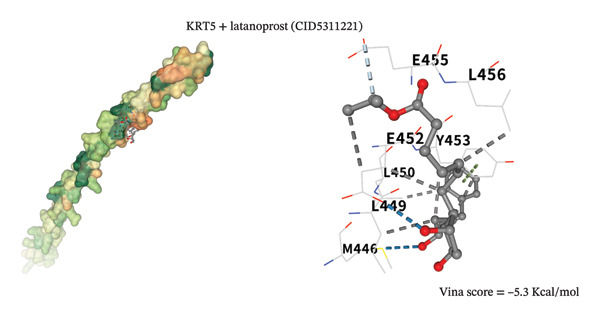
(c)

## 4. Discussion

POAG is a progressive neurodegenerative disease that affects more than 80 million individuals worldwide, leading to irreversible loss of vision due to retinal ganglion cell degeneration. Elevated IOP, primarily resulting from TM dysfunction, remains the only modifiable risk factor. However, currently available IOP‐lowering therapies fail to directly address the underlying cellular pathology of the TM, limiting their long‐term effectiveness [9, 17]. Recent studies have implicated dysregulation of autophagy, a critical lysosomal degradation pathway for maintaining cellular homeostasis, in the pathogenesis of POAG. Nonetheless, the specific molecular mechanisms linking autophagy dysfunction to disease progression are not fully elucidated [9]. In this study, we systematically analyzed transcriptomic data from human TM tissues, identified 15 DEATGs, constructed their regulatory networks, and revealed potential interactions between autophagy and immune dysregulation in glaucomatous TM. Among the DEATGs, S100A8 and S100A9 were consistently upregulated and functionally central, emerging as hub genes in the PPI network. Both genes belong to the S100 family of calcium‐binding proteins and form heterodimers known as calprotectin. Calprotectin plays diverse roles in both extracellular and intracellular contexts. Extracellularly, it contributes to antimicrobial defense, promotes inflammation, neutralizes reactive species, and induces apoptosis. Intracellularly, it supports cytoskeletal remodeling, pathogen resistance, and the activation of the NADPH oxidase complex, thereby increasing reactive oxygen species (ROS) production [18, 19]. ROS, in turn, are known regulators of autophagic flux. Supporting this mechanistic link, Ghavami et al. demonstrated that S100A8/A9 mediates mitochondrial membrane disruption via BNIP3 translocation, which enhances lysosomal activity, apoptosis, and mitophagy. Furthermore, this process promotes LC3 lipidation, reflected by the conversion of LC3‐I to LC3‐II, as well as Atg12 to Atg5 complex formation, both of which are essential steps in autophagosome maturation [20]. Additional studies have shown that S100A8/A9 modulates autophagy and apoptosis through the MAPK and PI3K AKT signaling pathways in cardiac tissues [21]. Clinically, the relevance of S100A8/A9 in glaucoma is supported by findings from Wong et al., who observed elevated tear levels of these proteins in medicated POAG patients compared to untreated individuals [22]. Our transcriptomic analysis adds to this evidence by identifying S100A8/A9 as molecular hubs in autophagy‐related dysfunction within the TM. Notably, we found a strong correlation between S100A9 expression and Th2 cell abundance. While previous studies have suggested immune involvement in POAG, our results specifically implicate S100A9 as a potential link between autophagy impairment and immune cell polarization in the TM microenvironment. Molecular docking simulations further predicted a high‐affinity interaction between latanoprost and S100A9, suggesting a possible pharmacologic mechanism by which prostaglandin analogs could influence autophagy–immune pathways in POAG. However, it is important to emphasize that this interaction remains a computational prediction, and experimental validation is required to determine its functional significance. Our functional enrichment analyses of DEATGs provide additional mechanistic insights. Subcellular localization analysis revealed predominant clustering within vesicular compartments, including secretory granule lumens, cytoplasmic vesicle lumens, and inclusion bodies (GO: 0034774, GO: 0060205, and GO: 0031983). This pattern is consistent with active involvement in autophagosome formation and maturation. MF annotations further indicated strong lipid‐binding capacity, particularly for long‐chain fatty acids and monocarboxylic acids (GO: 0036041, GO: 1901567, and GO: 0033293). These features suggest that DEATGs may participate in lipophagy, a selective form of autophagy that regulates membrane composition and vesicle elongation. Enrichment in BPs such as autophagy regulation and apoptotic signaling (GO: 0006914 and GO: 2001233) reinforces the hypothesis that DEATGs coordinate responses to cellular stress in POAG [23]. GSEA expanded the functional landscape of these genes, identifying three key pathological axes relevant to POAG. First, ECM remodeling and increased tissue stiffness were reflected in enrichment of pathways such as REACTOME_ECM_PROTEOGLYCANS and NABA_ECM_GLYCOPROTEINS. These changes are consistent with prior reports of ECM accumulation in the juxtacanalicular region, which contributes to AH outflow resistance and elevated IOP [24]. Second, DEATGs were enriched in metabolic pathways, including mitochondrial respiratory chain components (KEGG: 00190) and the tricarboxylic acid (TCA) cycle (KEGG: 00020), pointing to mitochondrial dysfunction and energy imbalance in TM cells. Third, stress and inflammatory signaling pathways, including VDR signaling (WP2872), interleukin‐1 mediated inflammation (PID: IL1_PATHWAY), the p53 pathway (BIOCARTA: P53_PATHWAY), and the ERK/MAPK cascade (KEGG: 04013), were also enriched. Together, these data suggest that autophagy dysfunction in POAG is not an isolated defect but a core disturbance linked to ECM dysregulation, metabolic decline, and chronic inflammatory signaling. Supporting this, studies have shown that active cellular metabolism is necessary for maintaining aqueous outflow in animal models, and that metabolic inhibition reduces outflow facility [25]. These findings align with epidemiological and molecular data connecting metabolic syndrome and POAG risk. A Mendelian randomization study identified type 2 diabetes as a causal factor in POAG development [26], while population‐based studies have shown higher glaucoma incidence among individuals with metabolic abnormalities [27]. Metabolomic analyses of AH, plasma, and TM tissue have revealed altered lipid and carbohydrate metabolism in POAG patients [28], and metabolic syndrome is known to impair autophagic function [29]. Additionally, vitamin D deficiency and polymorphisms in the VDR gene have been linked to POAG risk [30]. The VDR pathway influences autophagy through modulation of intracellular calcium levels, calcium‐dependent kinases, and regulators such as Bcl2 and mTOR [31]. Elevated IL‐1*α* and IL‐1β levels in POAG patient samples further support a proinflammatory environment [32]. However, IL‐1’s role in autophagy appears to be cell‐type and context dependent [33]. Although immune mechanisms in glaucomatous neurodegeneration have been recognized, the specific immune landscape within the TM has not been well defined. Our study provides a comprehensive characterization of immune cell infiltration in POAG TM, revealing a proinflammatory milieu with elevated levels of activated CD8+ T cells, macrophages, monocytes, NKT cells, pDCs, and Tregs. Conversely, we observed reduced levels of Th2 cells and neutrophils. These observations are consistent with prior reports of macrophage aggregation in glaucomatous TM [10] and altered T‐cell profiles in POAG patients [34]. Correlation analysis further supported the immunomodulatory role of S100A8 and S100A9. Both genes were positively associated with dendritic cell and CD56dim NK cell activation, indicating potential involvement in innate immunity [35, 36]. S100A9 was also correlated with CD4+ T cell and Th2 cell activation. Mechanistically, this is supported by studies showing that S100A8 activates dendritic cells and promotes Th1 polarization [37], while S100A9 has been implicated in dampening Th2‐driven allergic inflammation [38]. Given the ability of S100A8/A9 to modulate calcium signaling, a critical regulator of both autophagy and immunity, we propose that these proteins serve as dual mediators of autophagy–immune crosstalk in the glaucomatous TM. Furthermore, our docking analysis predicted a strong binding interaction between S100A9 and latanoprost, a widely used prostaglandin analog for IOP reduction. While the biological relevance of this interaction remains to be established, it raises the possibility that part of latanoprost’s therapeutic effect could involve modulation of S100A9‐mediated pathways, including autophagy and immune responses. These findings identify S100A8/A9 as candidate targets for future therapies aimed at restoring TM function through immune and autophagy modulation. Despite these promising findings, several limitations must be acknowledged. First, the transcriptomic nature of our study precludes causal inference. Although we identified DEATGs and hub genes associated with autophagy dysfunction, functional experiments are required to confirm their roles in autophagic flux. Second, the observed correlations between gene expression and immune cell infiltration require validation using coculture models of TM and immune cells, particularly under calcium‐regulated conditions. Third, although molecular docking suggested a high‐affinity interaction between S100A9 and latanoprost, experimental verification is necessary. Specifically, studies using latanoprost‐treated, S100A9‐overexpressing TM cells should be conducted to evaluate changes in autophagy markers such as LC3‐II and p62. Additionally, our study incorporated the GSE4316 dataset, which, while providing unique insights from microdissected human TM tissue, has a small sample size (*n* = 5). This is an inherent constraint of working with such rare clinical specimens. Although we employed rigorous statistical filters and cross‐validated our core findings with the larger GSE27276 dataset to ensure robustness, future studies utilizing larger independent cohorts or meta‐analyses of multiple datasets are warranted to further solidify our conclusions and enhance their generalizability.

## 5. Conclusion

This integrative, multidimensional analysis identifies S100A9 as a central hub gene that mediates autophagic deficiency in the TM of patients with POAG. Its expression is strongly correlated with Th2‐dominant immune dysregulation and shows high‐affinity binding to latanoprost, a widely used IOP‐lowering agent. These findings provide compelling evidence that S100A9 may serve as a mechanistic bridge linking impaired autophagy and immune dysfunction in the glaucomatous TM. Targeting this molecule could offer a novel therapeutic avenue to restore TM homeostasis by concurrently modulating autophagic activity and immune cell infiltration. Further experimental validation in preclinical models is warranted to confirm the functional role of S100A9 and to evaluate its potential as a therapeutic target in POAG.

## Author Contributions

Lili Hu, Shaoxin Pan, and Rui Chang conceived and designed the experiments, authored or reviewed drafts of the article, and approved the final draft. Lili Hu, Gong Chen, Min Lei, Ming Ai, Xiangyun Lv, Peng Wang, and Haoning Pan participated in the data analysis and preparation of figures and/or tables.

## Funding

This study was supported by the National Natural Science Foundation (82471062 and 82201167), Postdoctoral Fellowship Program of CPSF under grant number GZC20233346, China Postdoctoral Science Foundation (2025T180552), Chongqing Medical Youth Top Talent Program (YXQN202485), Wuhu Science and Technology Bureau Project (2025kj057), Natural Science Research Project of the Anhui Provincial Department of Education (Major Project: 2025AHGXZK20106 and Key Project: 2025AHGXZK31442), Scientific Research Startup Fund Project for High‐Level Talents of the Second Affiliated Hospital of Wannan Medical College (GRK202507 and GRK202518), and Clinical Medical Research Transformation Project of Anhui Province (202527c10020042).

## Disclosure

The manuscript has not been published in any other journal, nor has it been submitted or published as a preprint on any preprint server. All authors agree to submit and publish the manuscript in this journal. All authors have read and approved the final manuscript.

## Ethics Statement

This study does not contain any studies with human participants or animals performed by any of the authors.

## Conflicts of Interest

The authors declare no conflicts of interest.

## Data Availability

The datasets used in this study were downloaded from GEO. The ATGs were obtained from the GeneCards, GSEA databases, and PubMed. The R package used was obtained from Bioconductor. All data generated or analyzed during this study are included in this published article. If readers have any questions about the data processing, please do not hesitate to contact us (Lili Hu: lilihuhb@whu.edu.cn).

## References

[1] Martinez B. and Peplow P. V. , MicroRNAs as Biomarkers in Glaucoma and Potential Therapeutic Targets, Neural Regeneration Research. (November 2022) 17, no. 11, 2368–2375, 10.4103/1673-5374.338989. 35535873 PMC9120692

[2] Simcoe M. J. , Khawaja A. P. , Mahroo O. A. , Hammond C. J. , and Hysi P. G. , The Role of Chromosome X in Intraocular Pressure Variation and Sex-Specific Effects, Investigative Ophthalmology and Visual Science. (September 2020) 61, no. 11, 10.1167/iovs.61.11.20. PMC7490223 32926103

[3] Peng C. , Wu Y. , Ding X. et al., Characteristic Cytokine Profiles of Aqueous Humor in Glaucoma Secondary to Sturge-Weber syndrome, Frontiers in Immunology. (2020) 11, 10.3389/fimmu.2020.00004. PMC7008723 32117217

[4] Villasana G. A. , Bradley C. , Ramulu P. , Unberath M. , and Yohannan J. , The Effect of Achieving Target Intraocular Pressure on Visual Field Worsening, Ophthalmology. (January 2022) 129, no. 1, 35–44, 10.1016/j.ophtha.2021.08.025. 34506846 PMC10122267

[5] Cuervo A. M. and Macian F. , Autophagy, Nutrition and Immunology, Molecular Aspects of Medicine. (Feburary 2012) 33, no. 1, 2–13, 10.1016/j.mam.2011.09.001, 2-s2.0-84655164970. 21982744 PMC3996457

[6] Crawley O. , Opperman K. J. , Desbois M. et al., Autophagy is Inhibited by Ubiquitin Ligase Activity in the Nervous System, Nature Communications. (November 2019) 10, no. 1, 10.1038/s41467-019-12804-3. PMC6825199 31676756

[7] Hu X. , Lu Z. , Yu S. et al., CERKL Regulates Autophagy via the NAD-dependent Deacetylase SIRT1, Autophagy. (March 2019) 15, no. 3, 453–465, 10.1080/15548627.2018.1520548, 2-s2.0-85053876575. 30205735 PMC6351130

[8] Kasetti R. B. , Maddineni P. , Kiehlbauch C. et al., Autophagy Stimulation Reduces Ocular Hypertension in a Murine Glaucoma Model via Autophagic Degradation of Mutant Myocilin, JCI Insight. (March 2021) 6, no. 5, 10.1172/jci.insight.143359. PMC8021112 33539326

[9] Liton P. B. , The Autophagic Lysosomal System in Outflow Pathway Physiology and Pathophysiology, Experimental Eye Research. (March 2016) 144, 29–37, 10.1016/j.exer.2015.07.013, 2-s2.0-84951906468. 26226231 PMC4698029

[10] Taurone S. , Ripandelli G. , Pacella E. et al., Potential Regulatory Molecules in the Human Trabecular Meshwork of Patients With Glaucoma: Immunohistochemical Profile of a Number of Inflammatory Cytokines, Molecular Medicine Reports. (Feburary 2015) 11, no. 2, 1384–1390, 10.3892/mmr.2014.2772, 2-s2.0-84916210800. 25351602

[11] Yang J. , Patil R. V. , Yu H. , Gordon M. , and Wax M. B. , T Cell Subsets and sIL-2R/IL-2 Levels in Patients With Glaucoma, American Journal of Ophthalmology. (April 2001) 131, no. 4, 421–426, 10.1016/s0002-9394(00)00862-x, 2-s2.0-0035062239. 11292402

[12] Davis S. and Meltzer P. S. , GEOquery: A Bridge Between the Gene Expression Omnibus (GEO) and BioConductor, Bioinformatics. (July 2007) 23, no. 14, 1846–1847, 10.1093/bioinformatics/btm254, 2-s2.0-34547871639. 17496320

[13] Liu Y. , Allingham R. R. , Qin X. et al., Gene Expression Profile in Human Trabecular Meshwork From Patients With Primary Open-Angle Glaucoma, Investigative Ophthalmology and Visual Science. (September 2013) 54, no. 9, 6382–6389, 10.1167/iovs.13-12128, 2-s2.0-84884772799. 24003086 PMC3787658

[14] Liton P. B. , Luna C. , Challa P. , Epstein D. L. , and Gonzalez P. , Genome-Wide Expression Profile of Human Trabecular Meshwork Cultured Cells, Nonglaucomatous and Primary Open Angle Glaucoma Tissue, Molecular Vision. (July 2006) 12, 774–790. 16862071 PMC3152462

[15] Yu G. , Li F. , Qin Y. , Bo X. , Wu Y. , and Wang S. , GOSemSim: An R Package for Measuring Semantic Similarity Among GO Terms and Gene Products, Bioinformatics. (April 2010) 26, no. 7, 976–978, 10.1093/bioinformatics/btq064, 2-s2.0-77951942707. 20179076

[16] Yu G. , Wang L. G. , Han Y. , and He Q. Y. , ClusterProfiler: An R Package for Comparing Biological Themes Among Gene Clusters, OMICS. (May 2012) 16, no. 5, 284–287, 10.1089/omi.2011.0118, 2-s2.0-84860718683. 22455463 PMC3339379

[17] Porter K. , Hirt J. , Stamer W. D. , and Liton P. B. , Autophagic Dysregulation in Glaucomatous Trabecular Meshwork Cells, Biochimica et Biophysica Acta. (March 2015) 1852, no. 3, 379–385, 10.1016/j.bbadis.2014.11.021, 2-s2.0-84951906831. 25483712 PMC4312735

[18] Kerkhoff C. , Nacken W. , Benedyk M. , Dagher M. C. , Sopalla C. , and Doussiere J. , The Arachidonic Acid-Binding Protein S100A8/A9 Promotes NADPH Oxidase Activation by Interaction With p67phox and Rac-2, The FASEB Journal. (March 2005) 19, no. 3, 467–469, 10.1096/fj.04-2377fje, 2-s2.0-14644404879. 15642721

[19] Zhou J. , Li X. Y. , Liu Y. J. et al., Full-Coverage Regulations of Autophagy by ROS: From Induction to Maturation, Autophagy. (June 2022) 18, no. 6, 1240–1255, 10.1080/15548627.2021.1984656. 34662529 PMC9225210

[20] Ghavami S. , Eshragi M. , Ande S. R. et al., S100A8/A9 Induces Autophagy and Apoptosis via ROS-Mediated Cross-Talk Between Mitochondria and Lysosomes that Involves BNIP3, Cell Research. (March 2010) 20, no. 3, 314–331, 10.1038/cr.2009.129, 2-s2.0-77949270765. 19935772 PMC4161879

[21] Yi W. , Zhu R. , Hou X. , Wu F. , and Feng R. , Integrated Analysis Reveals S100a8/a9 Regulates Autophagy and Apoptosis Through the MAPK and PI3K-AKT Signaling Pathway in the Early Stage of Myocardial Infarction, Cells. (June 2022) 11, no. 12, 10.3390/cells11121911. PMC9221389 35741040

[22] Wong T. T. , Zhou L. , Li J. et al., Proteomic Profiling of Inflammatory Signaling Molecules in the Tears of Patients on Chronic Glaucoma Medication, Investigative Ophthalmology and Visual Science. (September 2011) 52, no. 10, 7385–7391, 10.1167/iovs.10-6532, 2-s2.0-84862833065. 21697136

[23] Marino G. , Niso-Santano M. , Baehrecke E. H. , and Kroemer G. , Self-Consumption: The Interplay of Autophagy and Apoptosis, Nature Reviews Molecular Cell Biology. (Feburary 2014) 15, no. 2, 81–94, 10.1038/nrm3735, 2-s2.0-84894565195. 24401948 PMC3970201

[24] Kasetti R. B. , Phan T. N. , Millar J. C. , and Zode G. S. , Expression of Mutant Myocilin Induces Abnormal Intracellular Accumulation of Selected Extracellular Matrix Proteins in the Trabecular Meshwork, Investigative Ophthalmology and Visual Science. (November 2016) 57, no. 14, 6058–6069, 10.1167/iovs.16-19610, 2-s2.0-84994741710. 27820874 PMC5102566

[25] Reina-Torres E. , Boussommier-Calleja A. , Sherwood J. M. , and Overby D. R. , Aqueous Humor Outflow Requires Active Cellular Metabolism in Mice, Investigative Ophthalmology and Visual Science. (August 2020) 61, no. 10, 10.1167/iovs.61.10.45. PMC7452856 32845955

[26] Shen L. , Walter S. , Melles R. B. , Glymour M. M. , and Jorgenson E. , Diabetes Pathology and Risk of Primary Open-Angle Glaucoma: Evaluating Causal Mechanisms by Using Genetic Information, American Journal of Epidemiology. (January 2016) 183, no. 2, 147–155, 10.1093/aje/kwv204, 2-s2.0-84960157897. 26608880 PMC4706681

[27] Jung Y. , Han K. , Park H. Y. L. , Lee S. H. , and Park C. K. , Metabolic Health, Obesity, and the Risk of Developing Open-Angle Glaucoma: Metabolically Healthy Obese Patients Versus Metabolically Unhealthy but Normal Weight Patients, Diabetes and Metabolism J. (June 2020) 44, no. 3, 414–425, 10.4093/dmj.2019.0048. PMC7332336 31950773

[28] Tang Y. , Shah S. , Cho K. S. , Sun X. , and Chen D. F. , Metabolomics in Primary Open Angle Glaucoma: A Systematic Review and Meta-Analysis, Frontiers in Neuroscience. (2022) 16, 10.3389/fnins.2022.835736. PMC9135181 35645711

[29] Madrigal-Matute J. and Cuervo A. M. , Regulation of Liver Metabolism by Autophagy, Gastroenterology. (Feburary 2016) 150, no. 2, 328–339, 10.1053/j.gastro.2015.09.042, 2-s2.0-84959470663. 26453774 PMC4728051

[30] Lv Y. , Yao Q. , Ma W. , Liu H. , Ji J. , and Li X. , Associations of Vitamin D Deficiency and Vitamin D Receptor (Cdx-2, Fok I, Bsm I and Taq I) Polymorphisms With the Risk of Primary Open-Angle Glaucoma, BMC Ophthalmology. (July 2016) 16, no. 1, 10.1186/s12886-016-0289-y, 2-s2.0-84978765261. PMC4952063 27435453

[31] Xu G. , Wang J. , Gao G. F. , and Liu C. H. , Insights Into Battles Between *Mycobacterium tuberculosis* and Macrophages, Protein and Cell. (October 2014) 5, no. 10, 728–736, 10.1007/s13238-014-0077-5, 2-s2.0-84930278551. 24938416 PMC4180456

[32] Wooff Y. , Man S. M. , Aggio-Bruce R. , Natoli R. , and Fernando N. , IL-1 Family Members Mediate Cell Death, Inflammation and Angiogenesis in Retinal Degenerative Diseases, Frontiers in Immunology. (2019) 10, 10.3389/fimmu.2019.01618, 2-s2.0-85069500027. PMC6646526 31379825

[33] Li W. , Jin D. , Hata M. et al., Dysfunction of Mitochondria and Deformed Gap Junctions in the Heart of IL-18-Deficient Mice, American Journal of Physiology—Heart and Circulatory Physiology. (August 2016) 311, no. 2, H313–H325, 10.1152/ajpheart.00927.2015, 2-s2.0-84983680284. 27288439

[34] Guo C. , Wu N. , Niu X. , Wu Y. , Chen D. , and Guo W. , Comparison of T Helper Cell Patterns in Primary Open-Angle Glaucoma and Normal-Pressure Glaucoma, Medical Science Monitor. (April 2018) 24, 1988–1996, 10.12659/msm.904923, 2-s2.0-85045418961. 29616680 PMC5900463

[35] Shaw J. , Wang Y. H. , Ito T. , Arima K. , and Liu Y. J. , Plasmacytoid Dendritic Cells Regulate B-Cell Growth and Differentiation via CD70, Blood. (April 2010) 115, no. 15, 3051–3057, 10.1182/blood-2009-08-239145, 2-s2.0-77951060089. 20139096 PMC2858470

[36] Vivier E. , Tomasello E. , Baratin M. , Walzer T. , and Ugolini S. , Functions of Natural Killer Cells, Nature Immunology. (May 2008) 9, no. 5, 503–510, 10.1038/ni1582, 2-s2.0-42449151214. 18425107

[37] Fujita Y. , Khateb A. , Li Y. et al., Regulation of S100A8 Stability by RNF5 in Intestinal Epithelial Cells Determines Intestinal Inflammation and Severity of Colitis, Cell Reports. (September 2018) 24, no. 12, 3296–3311 e6, 10.1016/j.celrep.2018.08.057, 2-s2.0-85053200419. 30232010 PMC6185744

[38] Palmer L. D. , Maloney K. N. , Boyd K. L. et al., The Innate Immune Protein S100A9 Protects From T-Helper Cell Type 2-Mediated Allergic Airway Inflammation, American Journal of Respiratory Cell and Molecular Biology. (October 2019) 61, no. 4, 459–468, 10.1165/rcmb.2018-0217OC, 2-s2.0-85072746376. 30943376 PMC6775951

